# A Bottom-Up Review of Image Analysis Methods for Suspicious Region Detection in Mammograms

**DOI:** 10.3390/jimaging7090190

**Published:** 2021-09-18

**Authors:** Parita Oza, Paawan Sharma, Samir Patel, Alessandro Bruno

**Affiliations:** 1 Computer Science and Engineering Department, School of Technology, Pandit Deendayal Energy University, Gandhinagar 382007, India; Paawan.Sharma@sot.pdpu.ac.in (P.S.); Samir.Patel@sot.pdpu.ac.in (S.P.); 2 Department of Computing and Informatics, Bournemouth University, Poole, Dorset BH12 5BB, UK

**Keywords:** mammograms, low-level features, machine learning, deep learning

## Abstract

Breast cancer is one of the most common death causes amongst women all over the world. Early detection of breast cancer plays a critical role in increasing the survival rate. Various imaging modalities, such as mammography, breast MRI, ultrasound and thermography, are used to detect breast cancer. Though there is a considerable success with mammography in biomedical imaging, detecting suspicious areas remains a challenge because, due to the manual examination and variations in shape, size, other mass morphological features, mammography accuracy changes with the density of the breast. Furthermore, going through the analysis of many mammograms per day can be a tedious task for radiologists and practitioners. One of the main objectives of biomedical imaging is to provide radiologists and practitioners with tools to help them identify all suspicious regions in a given image. Computer-aided mass detection in mammograms can serve as a second opinion tool to help radiologists avoid running into oversight errors. The scientific community has made much progress in this topic, and several approaches have been proposed along the way. Following a bottom-up narrative, this paper surveys different scientific methodologies and techniques to detect suspicious regions in mammograms spanning from methods based on low-level image features to the most recent novelties in AI-based approaches. Both theoretical and practical grounds are provided across the paper sections to highlight the pros and cons of different methodologies. The paper’s main scope is to let readers embark on a journey through a fully comprehensive description of techniques, strategies and datasets on the topic.

## 1. Introduction

Breast cancer is one of the most commonly diagnosed diseases amongst women worldwide. It is mainly detected on screening exams or the onset of clinical symptoms. Most breast cancers start in mammary glands [1]. The incidence of breast cancer has increased all over the world, and around one million new cases are reported every year [2]. Medical examinations are the most effective method for diagnosis of this cancer. Radiologists use various imaging modalities, such as mammography, breast MRI, ultrasounds, thermography and histopathology imaging. Visual inspections of images allow clinicians to identify suspicious areas that deserve further and more in-depth analysis. The visual inspection is an operator-dependent and time-consuming task. Over the last few decades, both academics and tech companies have proposed and developed proper computer-aided methods to assist the radiologist in diagnosing. Nowadays, CADe (computer-aided detection) and CADx (computer-aided diagnosis) systems are adopted as second opinion tools by expert clinicians for the detection of suspicious regions or abnormalities [3,4]. Most CADe and CADx tools rely on image analysis, machine learning (ML) and the deep learning (DL) approach.

Malignant and benignant masses are abnormal regions or cells that can be identified in mammograms. Various visual descriptors, such as shape, margin and density, are used to categorise abnormal cells. These descriptors are adopted in BI-RADS (Breast Imaging Reporting and Data System) [5], developed by the American College of Radiology. Shape and margin are adequate and discriminating descriptors for detecting masses [6]. For mammogram patch detection, low-level image features, such as interest keypoints, area, orientation, perimeter, and intensity, are frequently used [7,8]. Lot of work has been done to detect mammogram lesions using low-level image features, such as shapes, texture and local keypoint descriptors, which are discussed in this work.

AI (artificial intelligence) approaches, such as machine learning (ML) and deep learning (DL) gradually replaced these image processing-based techniques (e.g., methods relying on the analysis of low-level image descriptors, such as texture, local keypoints, and boundaries) because of their higher accuracy rates. Machine learning links the problem of learning from input data samples to the universal rules of inference. This approach uses analytical, statistical, and mathematical techniques that allow the machines to infer knowledge from training data without explicit programming. Some machine learning approaches [9,10,11], such as support vector machine (SVM), naïve Bayes, artificial neural network (ANN), and set classifiers [12], have become quite common for the development of computer-aided detection system for breast cancer. Machine learning techniques usually rely first on a step of image features’ extraction. Generally, the image features are described with arrays, namely descriptors, which feed training processes. The opportune choice of features then plays a fundamental role in the overall training accuracy. Historically speaking, there were some challenges motivating deep learning [13] that have represented an evolution in the traditional machine learning paradigm. Deep learning focuses on knowledge inference mechanisms from data and achieves higher levels of generalisation than in conventional machine learning. One of the most influential deep learning networks is the so-called CNN (convolutional neural network), characterised by convolutional layers. Other than traditional machine learning approaches, deep learning techniques are independent of feature extraction steps because of the high number of inner layers that somewhat perform feature extraction on the way through layer-embedded operators. DL-based algorithms are not trained to classify abnormal masses by inputting them with information about their shape, size, pattern, and other features; the algorithm itself learns what the mass looks like [14], using thousands of images during the training process. More details about techniques, architectures and models are provided in the corresponding sections of the paper.

Publicly available and adequately annotated datasets are rare in the medical imaging field; hence, there is a need for methods to deal with a low number of annotated images for training models and reaching a high accuracy rate. In this regard, two main approaches, such as transfer learning and unsupervised deep learning, turn out to be quite helpful. The former faces the lack of hand-labelled data, using pre-existing deep learning architectures and fine-tuning them onto a new application domain with a reduced number of samples [15]. The latter mainly derives direct perceptions from data and uses them for data-driven decision making. These approaches are more robust, meaning that they provide the base for varieties of complex problems, such as compression, classification, denoising, reducing dimensionality, etc. Unsupervised learning is also combined with supervised learning to create a model with added generalisation. Autoencoders and generative adversarial networks are widely adopted unsupervised deep learning approaches, which are discussed in the paper.

### 1.1. Motivation and Study Criteria

The main objective of this paper is to discuss different techniques in the literature to detect and/or classify suspicious regions spanning from mammograms using low-level image features to machine learning techniques and deep learning approaches. In the attempt to feed the open debate on the topic as mentioned earlier, the paper aims at answering the following questions:

- Which are various techniques to extract low-level image features from mammograms?
- What machine learning approaches tackle the detection of a mistrustful region in breast images?
- What are the various supervised and unsupervised deep learning approaches used for breast image analysis to detect and/or classify a suspicious region from a mammography image?
- What are the most commonly cited and publicly available mammogram datasets?

The survey also briefly discusses various forms of breast abnormalities—morphological features that are used by radiologists to detect suspicious masses and standard projection views of mammograms. This article further shows commonly cited and publicly available datasets of breast mammograms. The same datasets are compared. Furthermore, this paper mainly presents a comprehensive study of various methods in the scientific literature on the detection of suspicious regions from mammograms. Three main groups of methods are presented in this work: low-level image feature-based approaches, machine learning approaches, and deep learning approaches. The scientific literature is full of techniques that fall within each of these categories. One of the objectives of this paper is to discuss the most used and cited ones in the mammogram analysis domain.

This paper surveys hundreds of articles from indexed and referred journals, conference proceedings and books out of major online scientific databases, including IEEE Xplore, Web of Science, Scopus, and PubMed. Insightful and comprehensive surveys on mammographic image analysis are present in the scientific literature. Sadoughi et al. [16] thoroughly encompassed image processing techniques for detecting breast cancer by mostly focusing on artificial intelligence techniques. This paper aims to offer a bottom-up review, spanning both low-level image analysis and artificial intelligence techniques and providing the reader with all the materials needed to start working on the topic. For a more comparative analysis amongst studies, the paper is provided with relevant information, such as references, techniques used, scopes of work, datasets, and various performance metrics.

### 1.2. Paper Organization

**Figure 1:**
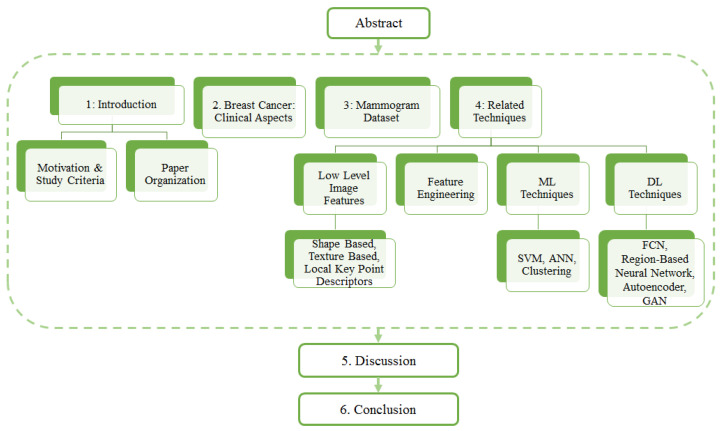
Organization of paper.

The overall structure of the paper is as follows. Section 2 provides readers with a description some clinical aspects of breast cancer in terms of mammogram projection views and various forms of breast abnormalities in mammograms. Section 3 provides an up-to-date list and details of mammogram datasets along with their comparisons. A link to the URL of each dataset is also provided. Section 4 reviews the related techniques, focusing on three categories and different approaches. Finally, the paper ends with a discussion Section 5, followed by a conclusion (Section 6). The organisation of the entire paper is depicted in Figure 1.

## 2. Breast Cancer: Clinical Aspects

### 2.1. Breast Positioning and Projection View

**Figure 2:**
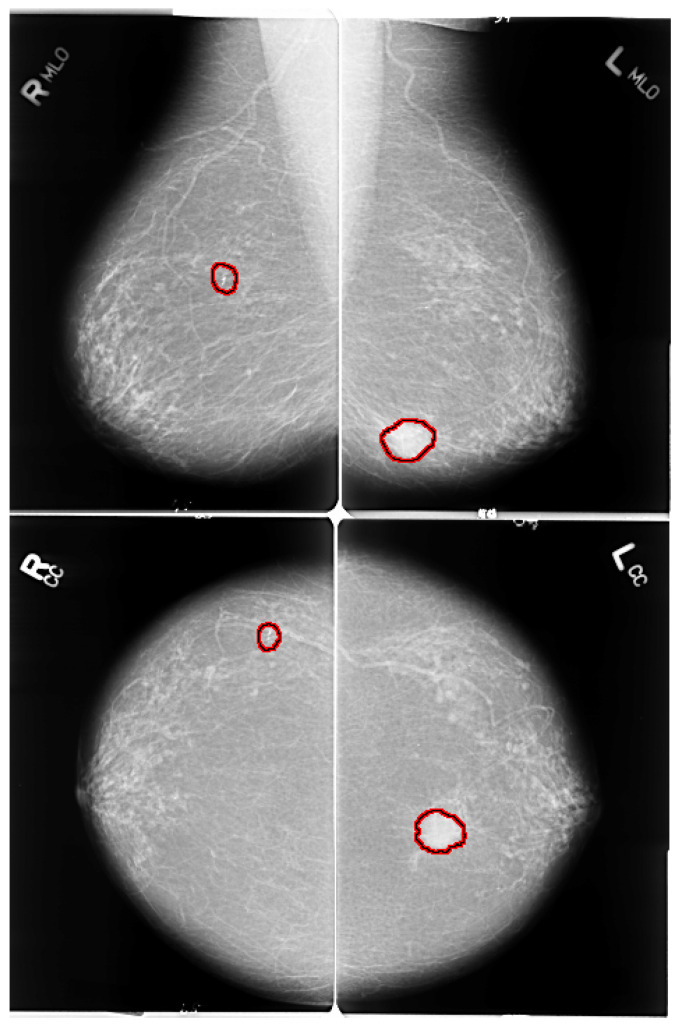
MLO and CC views of mammogram. Red highlighted sections in the images present abnormalities. Left images shows right MLO and CC views of benign calcification in upper outer quadrant of right breast. Right images shows MLO and CC views of spiculated mass lesion in lower inner quadrant of left breast.

The early detection of breast cancer depends on some crucial factors, such as the quality of the imaging technique and the patient’s position while the mammogram images are being taken. Breast positioning plays such a critical role in the process; improper positioning may result in inconclusive examination and mammogram artefacts. Mediolateral oblique (MLO) and bilateral craniocaudal (CC) represent the standard mammogram views. Both views encompass routine clinical screening mammography as depicted in Figure 2. It is essential to have proper and acceptable head-turning of the patient to obtain the CC view and raising of the arms of patients to obtain the MLO view. A correct CC projection should demonstrate the pectoral muscle on the posterior breast edge, maximum breast tissue and retro mammary space. As described by Moran et al. [17], a proper MLO view should ideally show the axilla, the tail of the axilla, and the inframammary fold along with the breast tissue. For an adequate breast cancer diagnosis, it is crucial to have multi-view mammographic data. Single-view mammograms may not provide enough information for a complete screening (some lesions might be missed). Andersson et al. [18] focused on the influence of the number of projections in mammography on breast disease detection. They reviewed 491 cases of breast cancer and evaluated the diagnostic importance of standard projection views. In their study, they reported that 90% of the malignancies were detected with a single projection view. The percentage of detected malignancies increased to 94% with multi-view projections. Furthermore, the latter reasonably lowers the number of false positives. Nowadays, many publicly available datasets include multi-view images [19].

### 2.2. Various Forms of Breast Abnormalities

**Figure 3:**
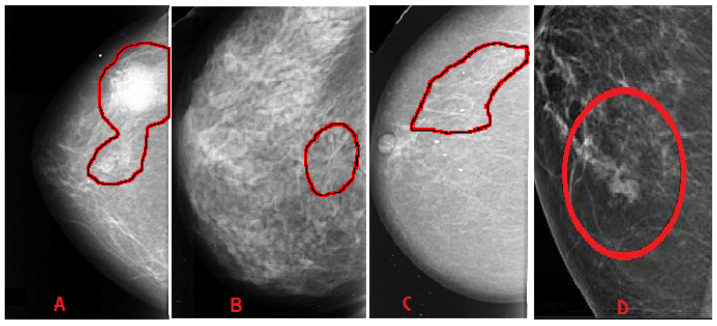
Categories of breast abnormalities. (**A**) Mass—well-defined irregular lesion, suspicious spiculated mass. (**B**) Architectural distortion. (**C**) Calcification—discrete microcalcification. (**D**) Asymmetry.

Breast abnormalities can assume different shapes and characteristics: mass (lesion), architectural distortion, calcification and asymmetry, as shown in Figure 3. These images are taken from publicly available mammogram datasets. This section briefly overviews these abnormalities and associated features.

**Figure 4:**
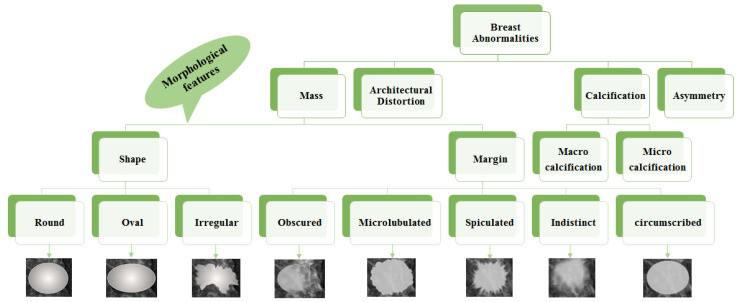
Taxonomy of breast abnormalities and morphological features in mammograms.

- Mass: A mass is a 3D lesion that can be seen in various projections. Morphological features, such as shape, margin and density, are used for mass characterisation. The shape can be round, oval or irregular. The margin can be not well defined, microlobulated, speculated, indistinct or circumscribed. Figure 4 shows the graphical representation of these morphological features (shape and margin) of a mass along with their subcategories. When superimposed breast tissues hide margins, that is called obscured or partially obscured. Microlobulated infers a suspicious finding. Spiculated margin with radiating lines is also a suspicious finding. Indistinct, also termed as ill-defined, is a suspicious finding too. Circumscribed is a well-defined mass that is a benign finding. Density can be high, low or fat-containing. The density of a mass is related to the expected attenuation of an equal volume of a fibroglandular tissue [6,20]. High density is associated with malignancy.
- Architectural distortion: This abnormality is found when normal architecture is distorted without certain mass visibility. Architectural distortion may include straight thin lines, speculated radiating lines, or focal retraction [6,20]. This abnormality can be seen as an additional feature. If there is a mass with distortion, it is likely to be malignant.
- Calcification: Calcifications are tiny spots of calcium that develop in the breast tissues. Arrangement of calcifications can be diffuse, regional, cluster, linear or segmental [6,20]. There are two types; macrocalcification and microcalcification. Macrocalcifications are large dots of white colour and often spread randomly within the breast area. Microcalcifications are small deposits of calcium, usually non-cancerous, but if visualised as particular patterns and clustered, they may reveal an early sign of malignancy.
- Asymmetries: These are the findings that show unilateral deposits of fibroglandular tissues, which cannot confirm the definition of mass. That can be seen in only one projection and is mainly caused by the superimposition of breast tissues that are normal [6,20].

Morphological features play an essential role in diagnosing breast diseases. Several studies evaluated the effectiveness of these features to diagnose the disease and to suggest the malignancy. Gemignani [21] presented a study on breast diseases. Mammographic lesions and microcalcifications are studied in the article. According to this study, masses with spiculated boundaries and irregular shapes have the highest chances of being carcinoma. Carcinoma is a common type of breast cancer. Rasha et al. [22] used morphological descriptors of BI-RADS for the characterisation of breast lesions. The study was carried out on a total of 261 breast lesions that were identified on contrast-enhanced spectral mammography in 239 patients. The authors concluded that morphological descriptors can be applied to characterise lesions. Most suggestive morphological descriptors are irregular-shaped mass lesions with spiculated and irregular margins. Wedegartner et al. [23] presented a study to check the expediency of morphological features to distinguish between malignant and benign masses. The result of the study shows that the irregular shape of the lesion is highly indicative of malignancy. The overall taxonomy of breast abnormalities and morphological features in mammograms are presented in Figure 4.

There is a well-defined tool for risk assessment and quality assurance, developed by the American College of Radiology, called BI-RADS (Breast Imaging-Reporting and Data System) [5]. Descriptors, such as shape and margin (along with their morphological features), are adopted in BI-RADS. Studies of breast imaging are allotted one of seven categories of BI-RADS assessment [24] as shown below:

- BI-RADS 0 (Assessment Incomplete)—Need further assistance.
- BI-RADS 1 (Normal)—No evidence of lesion.
- BI-RADS 2 (Benign)—Non-cancerous lesion (calcified lesion with high density).
- BI-RADS 3 (Probably benign) —Non-calcified circumscribed mass/obscured mass.
- BI-RADS 4 (Suspicious abnormality)—Microlubulated mass.
- BI-RADS 5 (High probability of malignancy)—Indistinct and spiculated mass.
- BI-RADS 6 (Proven malignancy)—Biopsy-proven malignancy (to check the extent and presence in the opposite breast).

Limitations of BI-RADS: The BI-RADS assessment is subjective. Several studies reported an anatomical variability in interpreting mammograms before the use of the BI-RADS lexicon, and it was not improved with the help of BI-RADS [25]. Beam et al. [26] conducted a study on the mammograms of 79 women, out of which 45 were cancerous. One hundred and eight radiologists reviewed these mammograms. The authors have reported that mammogram reading sensitivity and specificity varied from 47% to 100% and 36% to 99%, respectively. In another study, Berg et al. [27] presented intra- and inter-observer variability amongst five expert radiologists. The assessment of the lesions was highly variable. The readers agreed on only 55% of the total 86 lesions. Finally, Geller et al. [28] presented a study to check whether mammographic assessments and recommendations are appropriately linked or not as per BI-RADS. The study highlighted that BI-RADS 3 category had the highest variability.

## 3. Mammogram Datasets

This section briefs the publicly available mammography datasets that researchers use to detect and/or classify suspicious regions. Table 1 depicts a summary of the most cited and commonly used datasets. Sample images from these datasets are shown in Figure 3.

**Table 1:** List of commonly used mammogram datasets and reference URLs.

	Origin and Year	Total Cases	Total Images (Approx)	View Type	Image Type	Annotation	Reference Link for the Dataset
**SureMaPP**	UK, 2020	145	343	MLO	DICOM	Centre and radious of circle enclosing the abnormality	https://mega.nz/#F!Ly5g0agB!%E2%80%91QL9uBEvoP8rNig8JBuYfw (accessed on 27 October 2020)
**DDSM**	USA, 1999	2620	10000	MLO, CC	LJPEG	Pixel level boundary around abnormality	http://www.eng.usf.edu/cvprg/Mammography/Database.html (accessed on 31 May 2021)
**CBIS-DDSM**	USA, 1999	6775	10239	MLO, CC	DICOM	Pixel level boundary around abnormality	https://wiki.cancerimagingarchive.net/display/Public/CBIS-DDSM (accessed on 31 May 2021)
**INBreast**	Portugal, 2011	115	422	MLO, CC	DICOM	Pixel level boundary around abnormality	http://medicalresearch.inescporto.pt/breastresearch/GetINbreastDatabase.html (Link is taken from the base paper. Accessed on 31 May 2021)
**MIAS**		161	322	MLO	PGM	Centre and radious of circle enclosing the abnormality	https://www.repository.cam.ac.uk/handle/1810/250394 (accessed on 31 May 2021)
**BCDR**	Portugal, 2012	1734	7315	MLO, CC	TIFF	Unknown	https://bcdr.eu/information/about (accessed on 31 May 2021)
**IRMA**	Germany, 2008	Unknown	10509	MLO, CC	Several	Several	https://www.spiedigitallibrary.org/conference-proceedings-of-spie/6915/1/Toward-a-standard-reference-database-for-computer-aided-mammography/10.1117/12.770325.short?SSO=1 (accessed on 31 May 2021)
**BancoWeb** **LAPIMO**	Brazil, 2010	320	1473	MLO, CC	TIFF	ROI for few images	http://lapimo.sel.eesc.usp.br/bancoweb (assessed on 31 May 2021)

### 3.1. SureMaPP

SureMaPP [29] is a recently published dataset of mammograms with around 343 images manually annotated by experts in the field. Two different devices capture this dataset’s images: GIOTTO IMAGE SDL/W and FUJIFILM FCR PROFECT CS. Mammograms are available with two different spatial resolutions: 3584 × 2816 pixels and 5928 × 4728.

### 3.2. DDSM

The digital database of screening mammography (DDSM) [30] is a very old mammogram dataset. It consists of 2620 mammography studies from hospitals and medical universities in the U.S. Each case includes standard views, such as the mediolateral oblique (MLO) view and craniocaudal (CC) view for the left and right breast.

### 3.3. CBIS-DDSM

Curated Breast Imaging Subset of DDSM (CBIS-DDSM) [31] is a modified and standardised version of DDSM. Images of CBIS-DDSM are uncompressed and converted into DICOM format. This dataset includes an updated region of interest (ROI) segmentation and bounding box. Other pathological details, such as type of mass, grade of tumour and cancer stage, are included in the dataset.

### 3.4. INBreast

INBreast [32] has a total of 410 images acquired at the Breast Centre in CHSJ, Porto. As for CBIS-DDSM, DICOM format images with both MLO and CC views are provided. All images are annotated and validated by expert clinicians. Currently, Universidade do Porto has stopped supporting the dataset, but researchers may have access to the dataset by requesting the same.

### 3.5. MIAS

The Mammographic Image Analysis Society (MIAS) [33] dataset consists of 322 screening mammograms. Annotations are available in a separate file containing the background tissue type, class and severity of the abnormality, x and y coordinates of the centre of irregularities, and the approximate radius of a circle enclosing the abnormal region in pixels.

### 3.6. BCDR

The Breast Cancer Digital Repository (BCDR) [34] is a public mammogram dataset containing 1734 patient cases. These cases are classified as per Breast Imaging-Reporting and Data System (BIRADS). BCDR comprises two repositories: Film Mammography-Based Repository (BCDR-FM) and Full Field Digital Mammography-Based Repository (BCDR-DM). BCDR-FM contains 1010 patient cases with both MLO and CC views. BCDR-DM is still under construction. The BCDR dataset can be accessed by registering on the dataset website.

### 3.7. IRMA

The IRMA [35] dataset was developed from the union of various other datasets, such as DDSM, MIAS, the Lawrence Livermore National Laboratory (LLNL), and routine images from the Rheinisch-Westfälische Technische Hochschule (RWTH) Aachen. Images of this dataset are also available with both views. The dataset contains all types of lesions. IRMA is enriched with ROI annotations, which make the dataset suitable for supervised deep learning approaches.

### 3.8. BancoWeb LAPIMO

The BancoWeb LAPIMO [36] dataset is equipped with a total of 320 cases and 1473 images with MLO and CC views. Pictures of the dataset are grouped into the following categories: normal, benign and malignant. Annotations and patients’ background information are provided with BI-RADS. Annotations in the form of ROI are available for just a few images, while a textual description of the findings is available for all images. BI-RADS mammograms are in TIFF format.

## 4. Related Techniques

The scientific literature offers a wide variety of approaches for biomedical image processing tasks. Computer-aided detection (CADe) helps to detect regions of interest clinically from images. Computer-aided diagnosis (CADx) solutions measure the malignancy of masses [37]. CADe identifies suspicious regions and classifies a detected region as a mass or non-mass. CADx identifies a mass as a malignant or a benign one; it helps radiologists recommend biopsy or other follow-ups and treatment planning [38]. This section provides readers with a list of the state-of-the-art methods and techniques to detect suspicious regions from mammogram images. More precisely, it is noticed that most approaches in the scientific literature can be grouped into three prominent families. The following sections focus on methods relying on different techniques: the extraction of low-level image features, machine learning models, and deep learning models. For the sake of clarity, we want to highlight that in our work, we focus on methods for detecting suspicious regions in mammograms. However, we also consider those mammogram patch classification methods that have a model or architecture trained over small-sized image patches. We include them in this paper because those methods classify tiny areas of mammograms by labelling them as suspicious or non-suspicious. They return patches as classified, small enough to have them suitable for locating suspicious areas in the whole mammogram image.

### 4.1. Low Level Image Features

The detection of suspicious regions is accomplished with different approaches; the main focus in this subsection is to provide the reader with those techniques that rely on the extraction of low-level image features, such as shapes, texture and local keypoint descriptors. Furthermore, articles based on low level image features are summarized in Table 2 and Table 3.

#### 4.1.1. Shape Based Features

A non-negligible number of techniques for the detection of mammogram’s suspicious regions rely on the analysis of shape features, such as concavity, fractals, compactness, and morphological operators. The following subsections group shape-based feature analysis methods into two main categories: (1) techniques that rely on the numerical analysis of shape descriptors; and (2) techniques that employ shape descriptors to feed classification systems and neural networks.

##### Shape Descriptor Analysis Approaches

In this subsection, methods relying only on the numerical analysis of shape descriptors are presented. Raguso et al. [39] tackled the classification of breast masses by employing fractal analysis. Due to differences in shape complexity of breast masses contours, the fractal dimension is adopted as a discriminating feature for the purpose. Eltonsy et al. [40] reported that the growth of a mass forms concentric layers around activity areas in breast parenchyma structures; they proposed the so-called multiple concentric layers (MCLs) method to detect those activity areas. Morphological analysis of the concentric layer model is the cornerstone of the MCLs detection algorithm. There are three fundamental assumptions that guide the design of the MCLs algorithm. First, it is assumed that the focal activity layer is the brightest that and its evolving concentric layers tend to grow dimmer. Second, the morphology of the focal layer contains useful information regarding the presence of a potentially malignant mass. Third, if the relative incidence of a focal area with MCLs morphology is low in the breast region, then there is a higher probability that this area represents a true abnormality. Rangayyan et al. [41] tackled the detection of mammograms’ masses, using a boundary segmentation approach. They went first to separate major portions of the boundary and labelled them as concave or convex segments. Features were computed through an iterative procedure for polygonal modelling of the mass boundaries. The latter allows analysing shape features localised in each segment. Rangayaan et al. proposed a spiculation index based on the concavity fraction of a mass boundary and the degree of the narrowness of spicules. The spiculation index, fractional concavity and the global shape feature of compactness were combined for the boundary segmentation task. Chakraborty et al. [42] focused their efforts on the detection of the pectoral muscle from mammograms. The proposed method first approximates the boundary by a straight line. Within a selected region, maximum discontinuity points are determined along each horizontal line, based upon the weighted average gradient. An adaptive shape-based method is then applied to divide these points into a number of bands. The band with the maximum number of points is considered the most probable band containing probable pectoral edge points. A straight line is then estimated based upon the probable pectoral edge points. Surendiran and Vadivel [6] analysed the distribution of shape features, such as eccentricity, elongatedness, circularity1 (which measures how much a mass resembles a circle), circularity2 (which measures how much a mass resembles an ellipse), compactness, standard deviation, and dispersion to build on shape characteristics descriptors. Mustra et al. [43] proposed a method based on morphological operators and geometry for accurate nipple detection in craniocaudal mammograms.

##### Shape Descriptors for Classification Systems

This subsection aims to list and describe those classification systems fed with shape-based features. Li et al. [44] introduced a method to convert 2D breast mass’ contours in mammography into 1D signatures. The method provides a descriptor for contour features and breast mass regularity. The whole 1D signature is divided into subsections. The contour descriptor consists of four local features extracted from subsections. The contour descriptor is the root mean square (RMS) slope. On top of that, KNN, SVM and ANN classifiers are used to classify benign and malignant breast masses. Elmoufidi et al. [45] proposed a multiple-instance learning method based on the analysis of combined texture and shape features to classify malignant and benign masses in the breast. Elmoufidi et al. addressed the classification using features such as the equivalent circle of ROI (region of interest) and the bounding box, which is defined by the smallest rectangle containing the ROI. Zhang et al. [46] proposed a method that relies on two main steps encompassing eleven features: nine features from both spatial and morphology domains, and two cluster description features. The first stage plays a critical role in detecting coarse visual features, while the second stage allows for detecting finer analysis and classification. Soltanian-Zadeh et al. [47] ran microcalcification classification by comparing multi-wavelet, wavelet, haralick, and shape features. Several useful properties, such as symmetry, orthogonality, short support, and a higher number of vanishing moments, simultaneously can be retrieved by using the mentioned features. The usefulness of these properties is well known in wavelet design. The symmetric property allows symmetric extension when dealing with the image boundaries. Felipe et al. [48] carried out experiments to test out the effectiveness of Zernike moments as shape descriptors for mammograms’ classification. Zernike moments calculated from the pixels’ values of images retain pattern information related to shape. Soltanian-Zadeh et al. [49] presented two image processing methods for differentiating benign from malignant microcalcifications in mammograms. The gold standard method for differentiating benign from malignant microcalcifications is a biopsy, which is invasive. The goal of the proposed methods is to reduce the rate of biopsies with negative results. In the first method, the authors extracted 17 shape features from each mammogram. These features are related to the shapes of individual microcalcifications or to their clusters. In the second method, the co-occurrence method of Haralick achieves the extraction of 44 texture features from each mammogram. The best features from each set are extracted with a genetic algorithm by maximising the area under the ROC curve. This curve is created using a k-nearest neighbour (kNN) classifier and a malignancy criterion. The final step consists of comparing ROCs with the largest areas obtained, using each technique. Zyout et al. [50] presented a shape-based approach for the classification of MC (microcalcification) clusters. The proposed diagnosis scheme applies an embedded feature selection framework, called PSO-kNN (particle swarm optimisation and k-nearest neighbours), representing a unified scheme for accomplishing the feature selection and the classifier learning tasks. Sahiner et al. [51] dealt with the characterisation of breast masses on mammograms by developing a three-stage segmentation method based on clustering, active contour, and spiculation detection stages. After segmentation, extracted morphological features described the shape of the mass. Texture features were also extracted from a band of pixels surrounding the mass. Stepwise feature selection and linear discriminant analysis were employed in the morphological, texture, and combined feature spaces for classifier design. Junior et al. [52] proposed a method to detect breast cancer through diversity analysis, geostatistical and concave geometry (alpha shapes). Furthermore, they assessed the effectiveness of each feature, using support vector machine (SVM) in the MIAS and DDSM databases.

##### Pros and Cons

Some pros and cons for both approaches can be noticed by observing Table 2. Overall, those techniques based on the analysis of shape descriptors achieve good results in terms of accuracy. Raguso et al. [39] score 0.97 in the area under ROC. Nevertheless, the results are reported only on a local dataset, and no performance metrics are provided over publicly available databases. The experiments run by Rangayyan et al. [41] showed a certain level of accuracy and reliability of shape descriptors, such as spiculation index, fractional concavity and compactness on mass classification (accuracy of 80%) on MIAS. The method of Chakraborty et al. [42] reported very few false positives and false negatives in the detection of the pectoral muscle by using lightweight features, such as the average gradient. A morphological descriptor–based technique by Eltonsy, N.H. et al. [40] revealed high accuracy rates on DDSM, even though only the specificity is provided and no information about the number of false positives is given. Mustra et al. [43] used morphological descriptors and geometrical properties by which a 97.92% detection rate for nipple detection task was achieved in craniocaudal mammograms from a local dataset. The performance of the methods mentioned above shows reasonable accuracy rates and is lightweight. On the other side, their results show lower accuracy than the methods proposed by Li et al. [44], Zhang et al. [46], Sahiner et al. [51], and Junior et al. [52], which feed shape descriptors into SVMs, clustering, k-nearest classifier, etc. The method of Junior et al. showed a high sensitivity and detection rate on two different publicly available datasets (DDSM and MIAS), using geostatistical and concave geometrical descriptors and SVM.

#### 4.1.2. Texture-Based Features

Texture indicates visual patterns in visual content. Because of the complexity and diversity in natural images, texture analysis and description still represents a challenge [7]. In this section, the readers are provided with a description of texture analysis-based methods for detecting suspicious regions in mammograms. Like the previous section, this section is further divided into two subsections. The first one groups those methods relying on the analysis of texture feature descriptors, while the second one encompasses techniques, where the texture descriptors feed into the classification systems.

##### Texture Descriptors’ Analysis

Ramos et al. [53] proposed a technique that relies on texture features extracted from co-occurrence matrices, wavelet and ridgelet transform of mammogram images. They focused their efforts on detecting suspicious regions out of craniocaudal mammograms. Properties, such as entropy, energy, average, sum variance and cluster tendency, are calculated on top of texture features. The features mentioned above also undergo a GA (genetic algorithms) check to assess their effectiveness. Haindl and Remeš [54] dealt with enhancing suspicious breast tissue abnormalities, such as microcalcifications and masses, by helping radiologists detect developing cancer. They addressed the task, using a two-dimensional adaptive causal auto-regressive texture model to represent local texture characteristics. Furthermore, they used the Karhunen-Loeve transform to combine more than 200 local textural features (sensed with different frequency bands) into a single multi-channel image. Zheng et al. [55] presented a lattice-based approach that relies on a regular grid virtually overlaid on mammographic images. They used a local window centred on each lattice point to extract texture features within the breast. Tai et al. in [56], presented an automatic CADe (computer-aided detection) system that relies on local and discrete texture features for mammographic mass detection. In greater detail, Tai et al. [56] segmented adaptive square suspicious areas. In their study, the co-occurrence matrix and optical density transformation are used to describe suspicious regions with local texture and discrete photometric distributions. The same features undergo stepwise linear discriminant analysis to rank abnormal areas. Mudigonda et al. [57] introduced the concept of texture flow-field analysis to the mammogram analysis.

##### Texture Descriptors for Classification Systems

In the methods described in this subsection, texture descriptors feed into classifiers, logistic regression, clustering functions, and self-organising maps to classify suspicious regions in mammographic images. Farhan and Kamil [58] demonstrated the usefulness of texture analysis in mammograms to discriminate breast masses from normal tissue. They conducted their study on the MIAS [33], using LBP (local binary pattern) and a logistic regression classifier. Zheng et al. [55] as well as Mohanty et al. [59] extracted a range of statistical and structural (local binary pattern, and fractal dimension) features to carry out experimental detection of suspicious regions in mammograms. An association between lattice-based texture features and breast cancer was evaluated using logistic regression. Li et al. [60] presented a texture feature descriptor for mammographic image classification into different breast density categories. More precisely, they adopted the commonly used local binary patterns (LBP) and considered more feature details by including its variant, local quinary patterns (LQP). The descriptor turned out to be robust against rotations and translations. Quintanilla-Domínguez, et al. [61] analysed regions of interest (ROI) in mammograms by using the top-hat transform to enhance the ROI. Top-Hat transform increases the contrast between the background and well-defined/circumscribed masses, ill-defined masses, speculated masses, architectural distortions and asymmetries as described in MIAS [33]. After applying the top-hat transform with a 3×3 sized structuring element, the method carries out a window-based extraction of statistical moments, such as mean and standard deviation. K-means clustering and SOM (self organising maps) address grouping and labelling tasks. Hung and Lin [62] provided the scientific community with a GPU computing implementation of GLRLM to speed up the extraction process of texture features from mammograms. Gaussian smoothing and sub-sampling operations were employed to pre-process mammograms by Mudigonda et al. [57]. Mass segmentation is accomplished by establishing intensity links from the central portions of masses to their spatial neighbourhood. Mammogram regions are classified with flow orientation in adaptive areas of pixels across the boundaries of masses. A logistic regression system ingests the output of the last step. Biswas and Mukherjee [63] recognised architectural distortions in mammograms with a generative model carrying out the extraction of distinctive textures. The first layer of the architecture in this study consists of a multiscale-oriented filter bank that draws filter vectors as texture descriptors. The second layer of the proposed model is responsible for textural primitives’ representation tackled with a mixture of Gaussians.

##### Pros and Cons

Pros and cons of the previous section methods are described here. It is noticeable how texture descriptors, such as co-occurrence matrices, wavelet and ridgelet transforms [53], local texture features and KL transforms [54], lattice-based features [55], GLCM features and density features [56] and texture flow-field [57] perform reasonably well, in order, on mass classification, texture irregularity enhancement, density percentage in mammograms. Nevertheless, Ramos et al. [53] reach 0.9 in the AUC on a local dataset, while no performance metrics are given on public databases. The method of Haindl et al. [54] showed a remarkably high percentage of false positives. The method of Zheng et al. [55] achieved AUC values within the range [0.60, 0.74]. Tai et al. [56] obtained 99% sensitivity on DDSM but no data about false positives’ rates are given. With the only exception of local patterns [60], when texture features feed into classification systems [58,59,61], they return noticeably high rates of accuracy (93.6% on DDSM), sensitivity and specificity (greater than 99% on MIAS). However, as in the method of Biswas et al. [63], the classification systems’ performance may slightly drop when other texture features and descriptors are used (mixture of Gaussians).

#### 4.1.3. Local Keypoint Descriptors

Local keypoints and their corresponding descriptors, such as SIFT [64] and SURF [65], have been widely adopted over many computer vision topics [66]. Since their first introduction in 1999, a lot of water went under the bridge [64]. Different methods for image retrieval, image registration, object recognition, object classification, pattern recognition, robot localisation, wide baseline matching, and video shot retrieval ingest keypoint descriptors. Since then, biomedical researchers have started detecting regions of interest in images with local keypoint descriptors because of their robustness against varying illumination conditions and spatial noise distribution, geometric and photometric transformations in images. As far as it concerns the subject of this survey, several state-of-the-art methods rely on local keypoint descriptor. The following two subsections group methods and techniques into two main approaches: local keypoint descriptor analysis, and local keypoint descriptors for classification systems.

##### Local Keypoint Descriptor Analysis

Jiang et al. [67] proposed a method specifically for a query mammographic region of interest (ROI). They compared SIFT descriptors extracted from the ROI to a vocabulary tree, which stores all the quantised features of previously diagnosed mammographic ROIs. The method improves its performances with contextual information in the vocabulary tree. Jiang and Zhang conducted experiments on 11,553 mammographic ROIs. Guan et al. [68] focused on the liability of SIFT keypoints on microcalcification segmentation in MIAS dataset images. In greater detail, they fine-tuned the SIFT extraction parameters over MIAS by looking into the following steps: scale-space extrema detection, keypoint localisation, orientation assignment, and keypoint descriptor. Insalaco et al. [69] proposed a SURF-based method for detecting suspicious regions in mammograms. Their method is unsupervised and it relies on three main steps: pre-processing, feature extraction and selection. The histogram-based image intensity threshold assumes a relevant role in their method. The method allows extracting features from two versions of the same mammogram with different dynamic grey intensity levels.

##### Local Keypoint Descriptors for Classification Systems

In this section methods, local keypoint descriptors feed into classification systems. Utomo et al. [70] tested out some well-known scale and rotation invariant local features, such as SIFT, SURF, ORB, BRISK, and KAZE, to check which of them might replace the convolutional layers of CNN models. They kept the fully connected layers of the CNN model as the classifier, while they removed the convolutional layers. Furthermore, they assigned the high-level feature maps at the flatten layer with scale and rotation invariant local features. Utomo et al. [70] carried out experiments on MIAS dataset images. SIFT and SURF reportedly exhibited state-of-the-art performances when paired to VGG16 and MobileNetV2 models. Salazar-Licea et al. [71] presented a technique that combines SIFT features and K-means clustering to detect ROIs in mammograms. Their method first enhances the image quality through image thresholding and contrast limited adaptive histogram equalization (CLAHE). Then, they locate regions of interest in mammograms, using SIFT and binary robust independent elementary features (BRIEF) and feed keypoint coordinates into K-means clustering. Bosch et al. [72] presented a technique to model and classify breast parenchymal tissue. The authors analysed the spatial distribution of different tissue densities in an unsupervised manner by using SIFT and texture features. They also used both a classifier based on local descriptors and the probabilistic latent semantic analysis (pLSA) to detect meaningful aspects in mammograms. The MIAS and DDSM datasets were used to compare performances of different state-of-the-art methods to theirs. Liasis et al. [73] provided the scientific community with a method to investigate statistical distributions of different texture descriptors with support vector machines (SVMs) for breast density classification in mammogram images. They tested out features, such as SIFT, LBP (Local Binary Patterns) and texton histograms. TMatos et al. [74] conducted a study on the effectiveness of different features, such as (SIFT), speed up robust feature (SURF), oriented fast and rotated BRIEF (ORB) and local binary pattern (LBP) descriptors for local feature extraction in mammograms. The mentioned features are represented with the BOF (bags of features) approach to decrease data dimensionality. The same features fed support vector machine (SVM), adaptive boosting (Adaboost) and random forests (RF) for mammogram patch classification. Deshmukh and Bhosle [75] carried out some experiments on mammogram classification with SURF: they optimised SURF, ran the mammogram image classification with an associative classifier ingesting the optimised SURF. SURF descriptors and spatial distance measurements are taken into account to discard some regions. Mammogram classification relies on SURF features and the random forest method. Abudawood et al. [76] conducted experimental campaigns to assess LBP (local binary pattern) effectiveness against other image features, such as SURF, sparse coding and BRISK, using Gaussian process, k-nearest neighbours, support vector machines and AdaBoost.

##### Pros and Cons

**Figure 5:**
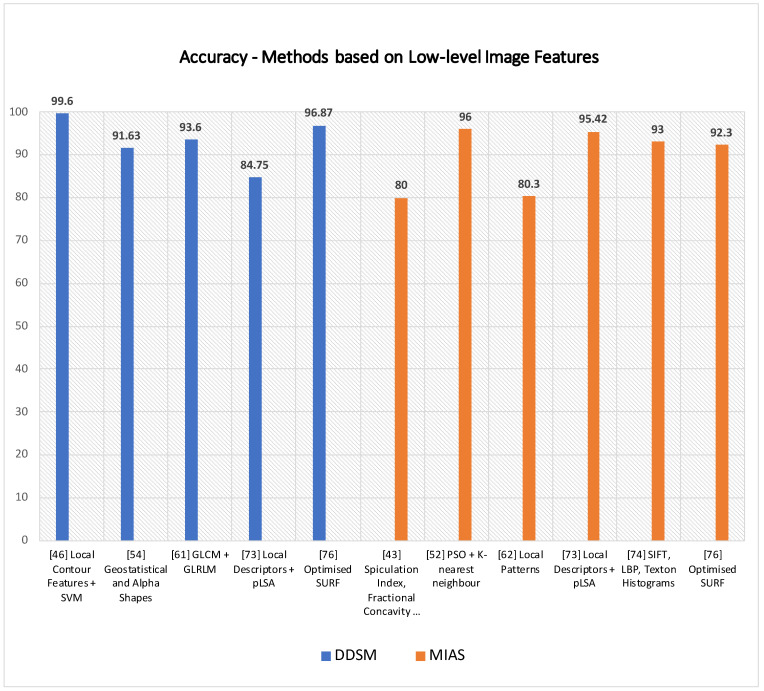
Some techniques based on the analysis of low-level features are plotted above according to their accuracy rates on DDSM and MIAS.

As for those methods based on shape and texture features, a further subsection provides the paper with the pros and cons of local keypoint-based methods. The method of Jiang et al. [67] successfully detected masses on a local mammogram dataset of 11,553 ROIs, achieving 90.8% accuracy by using an approach based on SIFT and vocabulary tree. It would be interesting to assess its performances on a public dataset to compare it with other state-of-the-art techniques on the task. SIFT descriptors were also adopted for detecting microcalcification by Guan et al. [68], but no accuracy metrics and comparison to other techniques are provided. Assuming that the method of Insalaco et al. [69] mainly relies on histogram-based image intensity thresholds, it would be interesting to know how robust the method is over datasets with different features. Utomo et al. [70] conducted noteworthy experiments to compare other keypoint descriptors feeding into FCN, and they achieved 100% sensitivity and specificity on MIAS for mass classification. It would be interesting to assess their performance on other datasets such as InBreast, DDSM and SuReMaPP. Bosch et al. [72] used local keypoint and pLSA for classifying parenchymal tissue on MIAS and DDSM. It is noticeable how performance accuracy rates drop by 13 percent from MIAS 95.42% to DDSM 84.75% showing better suitability of the proposed method for the MIAS dataset. Experimental results show higher accuracy rates for methods that feed local keypoint descriptors into classification systems at the expense of a higher computational cost. However, having most of the classification overall high accuracy performances, their accuracy rates drop when run over different datasets. In addition, the method of Deshmukh et al. [75] seems to have the most well-balanced accuracy rate over two datasets, such as DDSM and MIAS. It obtained, respectively, 92.30% and 96.87% accuracy rates on MIAS and DDSM. A graph showing accuracy rates of some methods based on low-level image features are shown in Figure 5. Due to heterogeneity in the metrics and datasets provided by the reference articles, only some techniques having the same datasets and metrics are plotted. More details are provided in Table 2 and Table 3 for a broader range of techniques.

**Table 2:** Methods based on low-level image features (a).

Reference	Technique	Task Performed	Dataset	Performances
[39]	Fractal Analysis	Mass Classification	Local Dataset San Paolo Hospital, Bari, Italy	Area under ROC: 0.97
[44]	Local contour features, +SVM	Mass Classification	DDSM	Accuracy: 99.6%
[45]	Multiple instance learning: textural and shape features + K-means	Mass Classification	DDSM and MIAS	Sensitivity: 95.6% on DDSM 94.78% on MIAS
[46]	Spatial and Morphology domain features	Microcalcification clusters’ detection	USUHS	Sensitivity: 97.6%
[47]	Multiwavelet, wavelet, Haralick, and shape features	Microcalcification classification	Nijmegen Database	Area under ROC: 0.89
[48]	Zernike moments	Classification of mammographic mass lesions	Local dataset	Precision: 80% Recall: 20%
[41]	Spiculation Index, Fractional Concavity, Compactness	Mass Classification	MIAS	Area under ROC: 0.82 Accuracy: 80%
[42]	Average Gradient and Shape Based Feature	Pectoral Muscle Detection	MIAS a local database	False Positives (FP) and False Negatives (FN): FP on MIAS 4.22%, FN on MIAS 3.93%;
[49]	Shape features and Haralick features.	Microcalcification classification	Nijmegen Database	Area under ROC: Shape Features 0.82; Haralick Features 0.72
[50]	Swarm optimisation (PSO) algorithm and k-nearest classifier	Microcalcification cluster detection	MIAS and a local dataset from the Bronson Methodist hospital	Accuracy: 96% on MIAS, 94% on BMH
[51]	Texture and Morphological Features	Mass Classification	local database	Area under ROC: 0.91 ± 0.02
[40]	Morphological Features	Mass Detection	DDSM	Sensitivity: 92%
[52]	Geostatistical and concave geometry (Alpha Shapes)	Mass Detection	MIAS and DDSM	Detection rate: 97.30% on MIAS and 91.63% on DDSM
[53]	Co-occurrence matrices, wavelet and ridgelet transforms	Mass Classification	Local Database	AUC = 0.90
[58]	Local Binary Pattern	Breast Mass Recognition	MIAS	Sensitivity 99.65% Specificity 99.24%
[54]	Local texture feature and KL Transform	Enhancing texture irregularities	Inbreast	True Positive 96% False Positive 65% False Negative 4%
[59]	GLCM and GLRLM features	Mass Classification	DDSM	Accuracy 93.6%
[43]	Pixel intensity and Morphological Features	Nipple detection	144 Mammograms (Local Dataset)	Detection Rate 97.92%

**Table 3:** Methods based on low-level image features (b).

Reference	Technique	Task Performed	Dataset	Performances
[55]	Texture Feature and Lattice Points	Mammographic Percent Density	Local Database	Area under Curve: 0.60–0.74
[60]	local patterns	Mass Classification	INBreast and MIAS	Accuracy: 82.50% on INBreat 80.30% on MIAS
[61]	morphological Top-Hat transform	mass and microcalcification detection	MIAS	Sensitivity and Specificity: 99.02% 99.94%
[62]	Texture Features analysis with GPU	Texture analysis in mammograms	DDSM and MIAS	CPU and GPU time on each picture
[56]	GLCM features and optical density features	Mass Detection	DDSM	Sensitivity 99%
[57]	Density Slicing and Texture Flow-Field Analysis	Mass Detection	MIAS	Area under Curve: 0.79
[63]	Mixture of Gaussian distribution for texture analysis in mammograms	Architectural Distortion Detection in Mammograms	MIAS and DDSM	MIAS Sensitivity 85.5% Specificity 81.0% DDSM Sensitivity 89.2% Specificity 86.7%
[70]	FC-VGG16 + SIFT, SURF, ORB, BRISK, and KAZE	Mass Classification	MIAS	SIFT + FC MobileNetV2 Specificity 100%; Sensitivity 100%;
[67]	SIFT features, Vocabulary Tree and Contextual Information	Mass Classification	Local dataset of 11553 ROIs from Mammograms	Accuracy 90.8%
[68]	SIFT features	Segmentation of Microcalcifications	MIAS	-
[71]	Scale-Invariant Feature and K-means clustering	ROI (Region of Interest) detection in mammograms	4 mammograms from MIAS	-
[72]	Local Descriptors and (pLSA)	Parenchymal Tissue Classification	MIAS and DDSM	Accuracy on MIAS 95.42%; DDSM 84.75%
[73]	SIFT, LBP and Texton Histograms and SVM	Breast Density Classification	MIAS	Accuracy 93%
[74]	Bag of Features (BoF) and SVM	Mass Classification	DDSM	Sensitivity 100% Specificity 99.24%
[69]	Histogram Specification and SURF features	Mass Detection	MIAS	Sensitivity 0.89
[75]	Optimised SURF	Mass Classification	MIAS and DDSM	MIAS Accuracy 92.30% DDSM Accuracy 96.87%
[76]	LBP plus classifiers (KNN, SVM, Gp, AB)	Abnormality Classification	DDSM	Precision 94.60% Recall 95%

### 4.2. Feature Engineering

**Figure 6:**
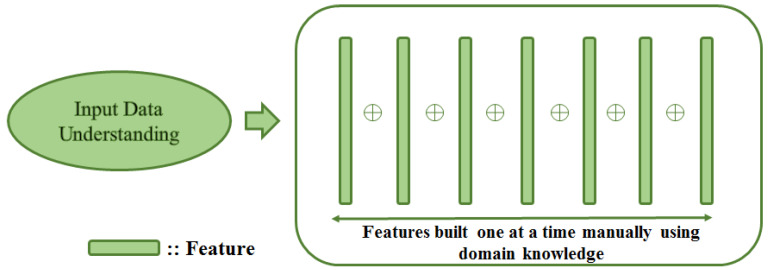
Manual feature engineering process: Features are crafted one at a time using domain knowledge by understanding underlying data.

Feature engineering allows formalising meaningful features from data. Machine learning models generally use these features for a variety of tasks. This technique transforms raw data into features that better represent the target to be learned. It features in the pipeline of many machine learning methods. That being said, feature engineering is specific to a problem and is error-prone. Moreover, finally crafted features are limited by user creativity. The manual feature engineering approach aims to create or build features one at a time with the help of domain knowledge as depicted in Figure 6.

The logic for crafting features is problem dependent and needs to change for every new application and underlying dataset. Feature engineering comprises various representative tasks, such as feature transformation, feature generation and extraction and feature selection. Feature engineering can be applied to all types of data and is frequently used with images and text. Chandakkar et al. [77] discussed various feature engineering aspects for image data, including texture, shape, colour features, etc. Some methods relying on these features are described in Section 4.1. In manual feature engineering, well-defined characteristics, such as texture, shape, and other low-level image features are constructed from input images to define imaging expression [78,79]. Though earlier researchers have used manually crafted features for various tasks, it is not always easy and possible to transform the medical imaging domain to precise scientific descriptors for manual feature engineering. This task becomes even more challenging when the field of application is not adequately understood [80].

When compared to deep learning, feature engineering needs pre-established descriptors or features of images or data. As far as it concerns medical images, this task is much more constrained because understanding this domain is mainly nonfigurative and difficult to define and set out with accurate scientific descriptors. With massive training data, deep learning automatically acquires inherent imaging features and uses these features to represent the outcome. Studies [80,81,82,83] show that feature extraction with the help of deep learning approaches presents better results as compared to pre-established descriptors in varieties of the application domain. An end-to-end deep learning model proposed by Arefan et al. [80] focuses on predicting short-term breast cancer risk using mammogram images. The authors used GoogleNet with the LDA model to extract deep features. As deep learning is considered a black box system, feature interpretation is not a straightforward process. Authors have used feature activation maps to highlight the essential and related regions in the images concerning the specific prediction tasks. Dhungel et al. [84] developed a method to learn the mammogram features automatically. Authors have used two-step training; pre-training based on the learning of a regressor that estimates the large set of handcrafted features that follow a fine-tuning stage that learns the classification of breast masses. It was shown in the study that the proposed method performs well when comparing to machine learning models with manually crafted features. Arora et al. [85] proposed a method for automatic classification of mammograms using ’deep features’. Features extraction and classification is carried out with deep ensemble learning and neural network classifier. The features that the ensemble model extracts are then optimised into a feature vector. Further, these features are classified with the help of neural network classifiers. The proposed model achieved an accuracy of 88%.

### 4.3. Machine Learning

In this section, several machine learning methods for classification and detection of suspicious regions are encompassed (see Table 4. In greater detail, methods relying on artificial neural networks, clustering techniques and classifiers such as SVM are tackled in the following subsections.

#### 4.3.1. Artificial Neural Networks

**Figure 7:**
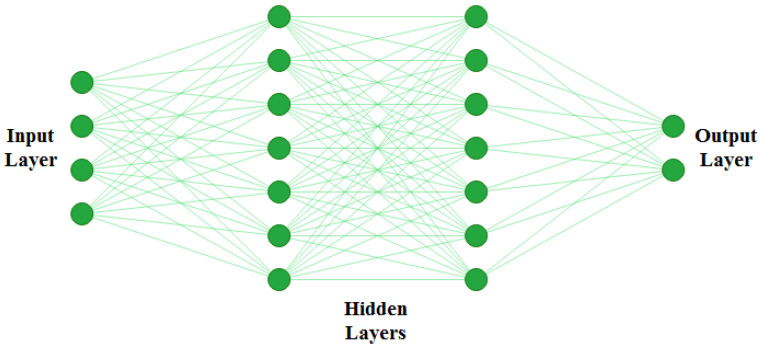
Basic structure of ANN. ANNs are generally defined as having three different layers; an input layer, hidden layer, and output layer. The input layer reads in data values from the user. There can be multiple hidden layers where actual learning takes place, and finally there is an output layer which presents the final result of the ANN.

The artificial neural network (ANN) is simply a computing system inspired by the biological neural network [86]. ANN works similarly to the biological neural networks of the human brain but does not resemble the same. The basic structure of ANN is depicted in Figure 7.

The functioning of ANN consists of two main steps: forward pass and backpropagation. During forwarding pass, feature values (Xi) are multiplied by weights (Wi), and the activation function is applied to each neuron. That generates a weighted output F(Xi). The next layer ingests F(Xi), and the process repeats till the output layer is reached. A typical ANN architecture can have one input layer, multiple hidden layers, and one output layer. Artificial neural networks are trained using backpropagation, which relies on the previous layer feedback. Finally, in the output layer, ground truth data are compared with the output generated by ANN. The loss function is responsible for determining the gap between results and labelled data. For updating weights, this loss is then transmitted to the network during the next iteration [87]. ANNs are among the most commonly used AI approaches for building CADe systems for mammogram analysis. It can be applied in different ways to mammograms; first, as a feature extractor and, second, as a classifier to directly classify the region of interest (ROI) of an input mammogram [88].

##### Artificial Neural Networks for Mammogram Analysis

In this subsection, ANN methods for detecting suspicious regions in mammograms are described. Wu et al. [89] inspected the potentiality of ANN as a decision-making tool to assist radiologist on mammogram analysis. In greater detail, the authors trained a three-layer feedforward neural network to analyse mammograms, using hand-labelled features by expert radiologists. Forty-three features allowed to reach 0.95 for the area under the ROC curve on lesion classification. An ANN-based system to analyse interpreted radiographic features from mammograms was proposed by Fogel et al. [90]. The model was trained with evolutionary programming to indicate the presence of malignancies. For suspicious masses, the model returned average area under ROC values of 0.91 with a specificity of 62% and sensitivity of 95%. Halkiotis et al. [91] proposed a model to detect clustered microcalcification using mathematical morphology and ANN. Morphological operators remove noise and regional maxima. The authors reviewed two models based on ANN; a two-layer MLP (multi-layer perceptron) and radial basis function neural networks (RBFNN) with variable hidden layers. With 10-hidden nodes, MLP could score a 94.7% positive detection rate and 0.27% false positives per image. Ayer et al. [92] presented an overview of ANN in mammography interpretation and diagnostic decision making and showed several limitations of existing ANN-based systems for detection and diagnosis. Quintanilla-Domínguez et al. [93] tackled the detection of microcalcifications using adaptive histogram equalisations and a self-organising map (SOM). Computer simulations confirmed the model’s capability and effectiveness to detect microcalcifications from mammograms. Papadopoulos et al. [94] proposed an approach for detecting and characterising microcalcification clusters from digitised mammograms. The method consists of three stages; cluster detection, feature extraction and classification that provides the final characterisation. The classification stage rule-based classifier, such as ANN and SVM, were evaluated using ROC analysis. The use of extra features extracted from the mammogram itself or collected from patient data could help the diagnostic process. García-Manso et al. [95] developed a system to detect and classify masses from mammograms. In this method, Independent Component Analysis (ICA) method is used to extract blind features, and neural networks run the classification process. Experimental results showed that the employment of heterogeneous datasets takes to variation in performance. Hupse et al. [96] developed an ANN-based CAD system to detect abnormal mass and architectural distortions from mammograms. The authors compared the results of human readers and the CAD system. The performance of the CAD system was close to that of certified experts in the field. One more CAD system for mammographic image feature analysis and screening is proposed by Tan et al. [97]. The system relies on ANN to predict the probability of a mammogram being positive. The authors used a ten-fold cross-validation method to test the performance of the system. The computed area under the ROC curve equalled 0.779 ± 0.025. Mahersia et al. [98] introduced a mass detection technique based on three steps: enhancement, characterisation and classification. The enhancement step helps to analyse breast texture. A Gaussian density function is used in the wavelet domain to segment the breast mass during the characterisation step. Last, a comparative classification method uses backpropagation networks and adaptive network-based fuzzy inference system (ANFIS) techniques.

#### 4.3.2. Clustering Techniques

Image segmentation is an essential task allowing the extraction of objects and their boundaries in a given picture. It is safe to say that segmentation plays such a fundamental role in medical image analysis. A properly segmented region helps practitioners retrieve vital information for the detection of diseases and may help them in surgical planning [99]. Clustering techniques are somewhat similar to classifier methods, except they do not use training data; hence they are called unsupervised methods. These methods iterate segmented images and characterize each class with properties to compensate lack of training data. K-means clustering relies on a simple algorithm, and it is moderately less computationally expensive. It is well suited to mammogram images because most breast regions can be clustered using pixel intensity values [100]. K-means algorithm partitions the dataset into *k* different clusters that are non-overlapping, and each data point fits into one cluster. Data points are assigned to clusters so that the total sum of the squared distance between the data points and the cluster’s centroid (mean of all data point of that cluster) is minimal. This approach helps to solve the expectation-maximization, where step *E* assigns the data points to the nearest cluster, and step *M* is to find clusters’ centroids. The objective function is as follows:

(1) $$J=\sum_{i=1}^{m}\sum_{k=1}^{K}w_{ik}{||x_{i}-\;\mu_{k}||}^{2}$$

where $w_{ik}$ = 1 for data point $x_{i}$ if it belongs to cluster *k*; Otherwise, $w_{ik}$ = 0 and $\mu_{k}$ is the centroid of $x_{i}$’s cluster

##### Clustering Techniques for Mammogram Analysis

A segmentation model was proposed by Kamil and Salih [101] to determine the boundaries of the tumour. The model builds on two algorithms based on clustering methods, such as K-means and fuzzy C-means. The authors employed a lazy snapping algorithm to improve model performances. After testing the model on the MIAS dataset, accuracy rates of 91.18% and 94.12% were achieved by K-means and fuzzy c-means algorithm, respectively. Ketabi et al. [102] presented a model to detect breast masses. It consists of the combination of three different approaches: clustering, texture analysis and support vector machine. Using data cleansing and greyscale enhancement, ROIs are detected and then segmented using spectral clustering. Features such as shape and probabilities are extracted from these segmented sections. For feature selection, the authors opted for genetic algorithms (GA). Finally, for the classification process, linear kernel was used. On the DDSM dataset, the classification accuracy obtained by the classification model is 90%. The segmentation of a region of interest from the image background is quite a complex task. The resulting segmented areas are generally pretty noisy. Kumar et al. [103] handled this issue by proposing a fuzzy c-means based model and incorporating similar superpixels and spatial information. Features of breast tissues such as the size of mass and size of breast ingested the model. The highest accuracy rate obtained was 84.32%. Hybridization of fuzzy c-means algorithm was proposed in [104]. Chowdhary and Acharjya et al. [104] addressed the co-presence of coincident clusters in the same mammogram with a possibilistic and intuitionistic fuzzy c-means.

#### 4.3.3. Support Vector Machine (SVM)

**Figure 8:**
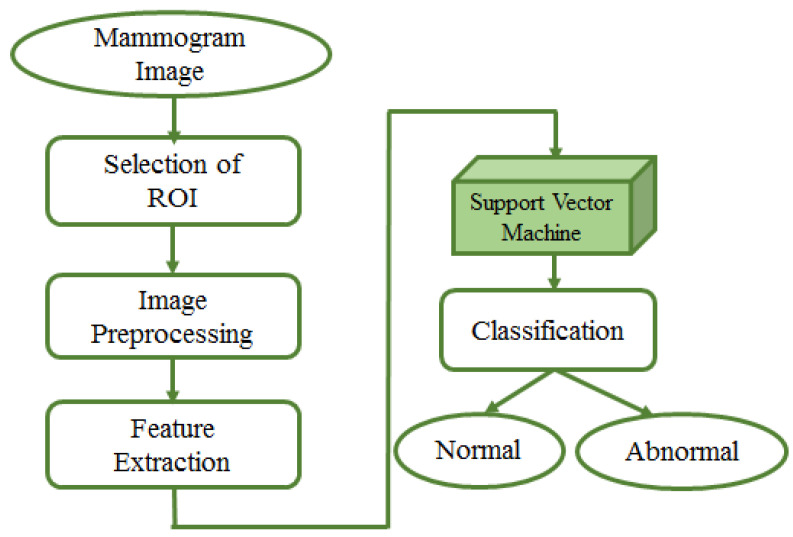
Mammogram image analysis by SVM. Input to the model is the mammogram image. Fixed-size region of interest (ROI) patches are extracted from mammogram images; pre-processing is done on extracted patches to improve the quality. Afterwards, a feature extraction step is needed to ingest SVM to train a model for classification of patches as benign or malignant.

In 1992, Boser et al. [105] presented a supervised algorithm for classification that successively evolved into the well-known support vector machines (SVMs) [106]. The key innovations of SVMs were the use of convex optimization, statistical learning theory, and kernel functions. The SVM method has been widely adopted in biomedical image analyses. Fixed-size region of interest (ROI) patches are extracted from mammogram images; then, patches are usually pre-processed for enhancement purposes. Afterwards, a feature extraction step is necessary to ingest SVM to train a model for classification. This entire process is presented in Figure 8.

The main idea of this approach is to find a line called hyperplane in *N* dimensions, which classifies the input data samples. A hyperplane is an (N−1)-dimensional subspace for an N-dimensional space. Mathematically, the hyperplane is defined as follows:

(2) $$w_{1}x_{1}+w_{2}x_{2}+\cdots +w_{d}x_{d}+\beta_{0}=0,$$

Here, *d* is the number of features, $x_{d}$ and $w_{d}$ are $d^{\mathrm{th}}$ feature and weights, respectively. $\beta_{0}$ is bias.

The main challenge of this approach is to find a plane called the maximum margin plane, which is necessary for accurate result prediction and generalised classifier. There is always an adjustment between correct classification and a large margin. If the margin is maximised to obtain better quality results, support vectors (data points closer to the plane) may be considered members of the other class. On the other way round, the margin could be narrow if we want an exact classification, which can lead to an improper or lower accuracy level of the model [107]. SVMs are widely adopted techniques for classification and regression task. SVM achieves very high accuracy rates when the data are linearly separable. For nonlinear data, kernel functions remodel nonlinear data to a high dimensional space; afterwards, SVM ingests the remodelled data. Varieties of kernel functions, such as linear, nonlinear, polynomial, and sigmoid are available for the purpose. When using SVM, the selection of kernel function and its parameters is a crucial challenge [108].

##### SVM for Mammogram Analysis

**Figure 9:**
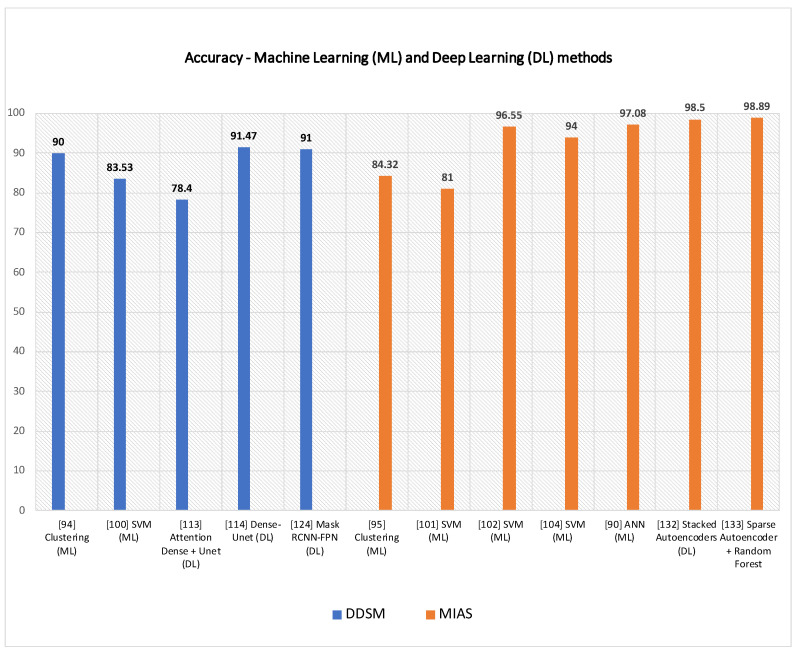
Some machine learning (ML) and deep learning (DL) techniques are shown above, according to their accuracy rates on DDSM and MIAS.

In this subsection, SVM-based techniques for mammogram analysis are described. Liu et al. [109] proposed the integration of possibilistic fuzzy C-means (PFCM) clustering and a weighted SVM to detect clusters of microcalcification regions. Suspicious regions are extracted using the region growing method and active contour segmentation. For each of these regions, texture and geometry features are obtained. For every sample, weights are calculated according to possibilities and typical values from the PFCM and the available ground truth. Suspicious regions of microcalcification are classified using weighted nonlinear SVM. This method was examined over around 410 images and compared with unweighted SVM. The performance of the model was evaluated based on ROC and FROC curve. The proposed approach could get better results as compared to standard SVM. A team of researcher presented a method in [110] for detecting masses from mammograms. Thresholding, correlation function and SVM tackled the detection process. Pre-processing increased the image contrast using a low pass filter. Wavelet transform with a linear function was used to enhance an image. The authors performed segmentation using thresholding. The post-processing step consisted of shape descriptors and SVM. Finally, SVM was again used for the classification process. Ancy and Nair [111] proposed a technique to detect breast cancer from mammograms using a gray level co-occurrence matrix (GLCM)–based SVM. After preprocessing a single view mammogram, ROI segmentation and GLCM feature extraction followed the classification process. This method was evaluated using two mammogram datasets named MIAS and University of South Florida Digital Mammography (USFDM). Qayyum and Basit [112] offered a model of breast cancer detection consisting of three main steps such as breast region segmentation (Otsus algorithm), pectoral muscle removal (using canny edge detection) and classification of normal and abnormal tissues (using SVM). Gray level co-occurrence matrices (GLCM) were used for feature extraction. The model was evaluated on the MIAS dataset. Results compared to previously proposed methods showed the reliability of the method of Qayyum et al. A CAD to detect suspicious areas in mammograms and classify them as benign or malignant was proposed by Sharma and Khanna [113]. The preprocessing step fulfilled the background breast region separation. Zernike moments of different orders described fixed-size (128 × 128) patches extracted from input mammograms as feature vectors. SVM eventually classified the extract ROI patches that. The experimental result showed better results obtained with order 20 Zernike moments and an SVM. To detect mammogram image features, Vijayarajeswari et al. [114] used Hough transform, and these features are used as an input to the SVM classifier. This model achieved an accuracy of 94%, which was very good when compared to the accuracy obtained by the LDA classifier (86%). Findings from various articles based on Machine Learning approaches for breast cancer diagnosis are summarised in Table 4. Some techniques are also compared in Figure 9 Due to the heterogeneity of datasets and metrics reported in the reference articles, only the accuracy rates of machine learning and deep learning over DDSM and MIAS are plotted in Figure 9.

**Table 4:** Machine learning approaches for breast cancer diagnosis from mammograms.

Reference	Technique	Task Performed	Dataset	Model Performace
[101]	Clustering	Mass Segmentation	MIAS	K-means: 91.18% Fuzzy c-means: 94.12%
[102]	Clustering	Mass Detection	DDSM	Accuracy: 90%
[103]	Clustering	Suspicious Lesion Segmentation	MIAS	Accuracy: 84.32%
[109]	SVM	Microcalcification Detection	InBreast	ROC: 0.8676 Sensitivity: 92% FPR: 2.3 clusters/image
[110]	SVM	Mass Detection, Mass Classification	DDSM	Sensitivity: 92.31% Specificity: 82.2% Accuracy: 83.53% ROC: 0.8033.
[111]	SVM	Tumor Detection	USFDM, MIAS	Precision:0.98 Sensitivity: 0.73 Specificity: 0.99 Accuracy: 0.81 Score: 0.758
[112]	SVM	Segmentation, Classification	MIAS	Accuracy: 96.55%
[113]	SVM	Abnormality Detection	IRMA, DDSM	IRMA: Sensitivity: 99% Specificity: 99% DDSM: Sensitivity: 97% Specificity: 96%
[114]	SVM	Mammogram Classification	MIAS	Accuracy: 94%
[89]	ANN	Lesion Classification	Mammography Atlas	ROC: 0.95
[90]	ANN	Mammogram Feature Analysis	Private	ROC: 0.91 Specificity: 62% Sensitivity: 95%.
[91]	MLP, RBFNN	Microcalcification Detection	MIAS	Positive detection rate: 94.7% False positives per image: 0.2%
[94]	SVM, ANN	Microcalcification Characterization	MIAS	SVM: Original feature set, Az: 0.81 Enhanced feature set, Az: 0.80 ANN: Original feature set, Az: 0.73 Enhanced feature set, Az: 0.78
[95]	ANN	Detect and Classify Masses	DDSM	AUC = 0.925
[96]	ANN	Detection of Mass and Architectural Distortion	Private	TPF: 0.620
[97]	ANN	Detection of Breast Cancer	Private	AUC = 0.779 ± 0.025
[98]	ANN	Mass Detection	MIAS	Recognition Rate = 97.08%

##### Pros and Cons of Machine Learning Approaches

The previous sections encompassed several machine learning methods, such as clustering, ANN and SVM, accomplishing computer vision tasks over mammograms. Here pros and limitations of those techniques are stressed out to enrich the narrative with some considerations and experimental details. Models’ performances are reported in Table 4 according to tasks performed, techniques adopted, and datasets used. For example, the methods of Kamil et al. [101] and Kumar et al. [103] proved the highest accuracy rates, respectively, with 94.12% and 90% over MIAS and DDSM among the clustering-based methods. The ANN-based method proposed by Fogel et al. [90] reached a high sensitivity rate of 95% on a private dataset. At the same time, specificity is lower by 33%, showing a not negligible number of false positives. Conversely, the ANN-based methods by García-Manso et al. [95], and Mahersia et al. [98] achieved, respectively, 0.925 in AUC on DDSM and ranked the highest recognition rate of 97.08% on MIAS. Overall, the best performances over IRMA and DDSM datasets are shown by SVM in the method proposed by Sharma and Khanna [113]. They obtained, respectively, 99% of sensitivity and specificity on IRMA, and 97% sensitivity and 96% specificity on DDSM. Qayyum and Basit [112] reached 96.55% accuracy in both segmentation and classification of suspicious regions over MIAS. Overall, supervised machine learning approaches such as SVM and ANN outrank clustering techniques in the detection of suspicious areas on mammograms, even though the fuzzy c-means–based method by Kumar et al. [103] achieves up to 94.12% accuracy on MIAS.

### 4.4. Deep Learning Approaches

#### 4.4.1. Supervised Deep Learning

Other than conventional machine learning approaches, newer AI approaches called deep learning (DL) [13] are playing a critical role in the progress of many scientific fields. Unlike the more traditional machine learning techniques, DL architectures are fed with raw data and automatically build those data representations necessary for detection or classification. Deep learning architectures consist of several simple but non-linear modules, which process the representation at different levels, spanning from the raw input to increasingly higher representation layers. Deep learning methods, figuratively speaking, are like complex algebraic circuits whose connections can be fine-tuned. The word deep takes after the many layers characterising architectures. Deep also means that computations across the whole architecture pipeline have many steps. Deep learning is currently the most widely used approach for visual object recognition, machine translation, speech recognition, speech synthesis, and image synthesis. Over the last decades, AI researchers extended pre-existing architectures and proposed some new ones. Fully convolutional networks (FCN), region-based CNN (R-CNN), faster R-CNN, and mask R-CNN, ResNet, inception, VGGNet, feature pyramid networks, cascade R-CNN and libra R-CNN are just some of the most popular architectures adopted for classification and detection tasks. This section discusses deep learning–based approaches for the detection of suspicious regions in mammograms. Table 5 summarises articles based on both supervised and unsupervised deep learning approaches for breast cancer diagnosis.

##### Fully Convolutional Network (FCN)

FCN was proposed in [115] is characterised with exclusively locally connected layers like convolutional, pooling and upsampling across encoder and decoder branch. By avoiding dense layers, this network reduces the number of parameters making training faster. FCN also includes downsampling (encoder) and upsampling (decoder) path for extracting and interpreting an image and localisation context, respectively. This network can work with input images of any size and returns output with equal input dimensions. The network also adopts the concept of skip connection to recover the loss of fine-grained spatial information during the downsampling path.

**Table 5:** Deep learning approaches for breast cancer diagnosis.

Reference	Technique	Task Performed	Dataset	Model Performace
[116]	FCN	Breast Density Estimation	Private	Pearson’s rho values: CC View: 0.81 MLO View: 0.79
[117]	FCN	Mass Segmentation	DDSM, INBreast	DDSM: Dice similarity coefficient: 0.915 ± 0.031 Hausdorff distance: 6.257 ± 3.380 INBreast: Dice similarity coefficient: 0.918 ± 0.038 Hausdorff distance: 2.572 ± 0.956
[118]	FC-Densenet	Tumor Segmentation	Private	Dice Index: 0.7697 Pixel Accuracy: 0.7983 Intersection Over Union: 0.6041
[119]	Unet	Mass and Calcification Detection	CBIS-DDSM, INBreast	MassDice score: 67.3% Sensitivity: 70.3%
[120]	Attention Dense—Unet	Mass Segmetation	DDSM	F1 Score: 82.24 ± 0.06 Sensitivity: 77.89 ± 0.08 Specificity: 84.69 ± 0.09 Accuracy: 78.38 ± 0.04
[121]	Dense-Unet	Calcification Detection	CBIS-DDSM	Accuracy: 91.47% Sensitivity: 91.22% Specificity: 92.01% F1 Score: 92.19%
[122]	CSA Block, Cascade RCNN	Mass Detection	Private, CBIS-DDSM	Average precision: 0.822 Average recall: 0.949
[123]	Faster RCNN	Mass Detection	INBreast, Private	TPR—0.88 FPs/I—0.85
[124]	Faster RCNN	Mass Detection	OMI, INBreast	TPR at FPI: OMI-H: 0.93 at 0.78 OMI-H OMI-G: 0.91 ± 0.06 at 1.70 Inbreast: 0.92 ± 0.08 at 0.30 0.85 ± 0.08 at 1.0 0.95 ± 0.03 at 1.14
[125]	RCNN	Architecturak Distrotion Detection	DDSM	Sensitivity and specificity: 80% FPI: 0.46, TPR: 83%
[126]	Faster RCNN	Mass Detection	DDSM	Average Precision: Inception ResNet V2: 0.85
[127]	Mask RCNN-FPN	Multi Detection and Segmentation of Breast Lesions	DDSM, INBreast	Overall Accuracy: 91%
[128]	Faster RCNN	Mass Detection	Private	AUC: 0.96
[129]	Faster RCNN	Detection and Classification of Mammogram Lesions	INBreast	AUC: 85%
[130]	GAN, ResNet	Data Augmentation, Mammogram Classification	DDSM	AUC: 0.896
[131]	GAN, U-Net	Data Augmentation, Classification	OMID	AUC: 0.846
[132]	GAN	Mass Image Synthesis	DDSM, Private	AUC DDSM: 0.172 Private: 0.144
[133]	CycleGAN	Mass Image Synthesis	BCDR, INBreast	-
[134]	GAN	Mammogram Synthesis	Private	-
[135]	Sparse Autoencoder	Breast Density Segmentation	Private	PMD scores on AUC: 0.59
[136]	Sparse Autoencoder	Breast Asymmetry Analysis	Private	Sensitivity: 0.97
[137]	Denoising Autoencoders	Breast Density Scoring	Private	AUC: 0.68
[138]	Stacked Autoencoders	Mammogram Classification	MIAS	98.50%
[139]	Sparse Autoencoder, ML classifiers	Mass Classification	MIAS	Accuracy by Random forest: 98.89%
[140]	Autoencoder	Mammography Classification	INBreast, IRMA	Accuracy: 98.45%

##### FCN for Mammogram Segmentation

FCN is mainly employed for semantic segmentation and widely adopted by the research community for breast cancer diagnosis and segmentation. Lee and Nishikawa [116] presented a fully automated deep learning–based system to estimate breast density from mammograms. A fully convolutional network is used for the segmentation of breast and dense fibroglandular areas. The model results are compared with the state-of-the-art algorithm called the laboratory for individualised breast radio density assessment (LIBRA). Breast percentage density (PD) and dense area fraction are estimated by the model correlated with the Breast Imaging Reporting and Database System (BI-RADS) assessment strategy. The proposed model also showed notable results to separate each BI-RADS breast density class where LIBRA failed. A multichannel and multiscale fully convolutional network for mammogram mass segmentation was proposed by Xu [117]. Preprocessing was carried out to reduce the influence of nearby structures that are negligible. Multi-scale features are learnt from various resolution levels for obtaining smooth boundary and global mass localisation. The model was evaluated and compared on two publicly available datasets: DDSM and INBreast. Indexes such as the Dice similarity coefficient and Hausdorff distance revealed the method’s performance. Values obtained for these measures were 0.915 ± 0.031 and 6.257 ± 3.380 on DDSM and 0.918 ± 0.038 and 2.572 ± 0.956 on INbreast, respectively. Hai et al. [118] set up an end-to-end fully convolutional network to segment breast tumour. Hai et al. [118] addressed diversity in shape and size of abnormal tumours by introducing multiscale image information into the fully convolutional dense network. The utilisation of a weighted-loss function tackled the problem of a not well-balanced class image number. The absence of preprocessing and post-process steps makes the system lighter weight. A method to detect abnormalities like mass and calcification was proposed by Sathyan [119]. These abnormalities are segmented using a fully convolutional architecture called Unet [141]. This model for mass segmentation and calcification segmentation is trained on the CBIS-DDSM and INBreast datasets, respectively. A fully automated deep learning–based method was proposed by Li et al. [120] using densely connected U-Net along with attention gates (AGs). The system includes an encoder–decoder pair. U-Net consists of an encoder (densely connected convolutional network) and a decoder branch (an attention gate in this case). Performance parameters, such as sensitivity, specificity, Fi-score, etc., allowed the authors to evaluate and compare their method to state-of-the-art methods. AlGhamdi et al. [121] developed a model to detect breast arterial calcifications using U-Net with dense connectivity. This model allows the reuse of computation that is already done and improves the gradient flow, leading to better model accuracy.

##### Region Based Neural Networks

The latest research emphasises improvements in feature extraction, feature selection and feature classification capabilities of CNNs. CNN instances, such as RCNN, Fast RCNN and Faster RCNN lay on the so-called region proposals. Region proposals are tiny image regions containing objects of interest (the object of interest depends on the application domain). It helps to predict probable object positions from the image. The selective search approach is commonly used to generate region proposals. This section provides insight into such region-based networks.

##### Region Based Convolutional Neural Networks (R-CNN)

R-CNN was proposed by Girshick et al. [142] in 2013. It builds on a selective search approach used to generate thousands of region proposals. Selective search is a greedy method combining small segmented regions for generating region proposals. A CNN ingests 2000 region proposals for computing features ingested by SVM to classify the object of interest. However, RCNN presents some limitations.

- The approach is not suitable for real-time applications because of its computational cost.
- Selective search approach is not flexible; no learning takes place in it.
- Training happens in three phases; CNN fine-tuning, SVM training and bounding box regressor on thousands of candidate proposals.
- For all region proposals, it is necessary to save feature maps that need a large amount of memory space during training.

##### Fast RCNN

Girshick [143] designed fast RCNN to solve the computational load of RCNN architectures and detect the object of interest (suspicious regions in mammograms). In Fast R-CNN, selective search generates 2000 region proposals from the input image as in R-CNN. All the region proposals, alongside the input image, are then passed onto a CNN for generating feature maps. For each of these region proposals, the region of interest pooling layer extracts fixed-size feature vectors. Layers of softmax and bounding box regressor ingest the extracted feature vectors for the classification of region proposals. Fast R-CNN is based on pre-trained networks (trained on ImageNet dataset, such as the VGG-16 network) with 5 to 13 convolutional layers and five max-pooling layers. A summary of the overall architecture is given below:

- The CNN architecture takes image (size = 224×224×3 for VGG-16) and its region proposal and outputs the convolution feature map (size = 14×14×512 for VGG-16).
- Last pooling layer (layer before fully connected layer) is replaced with a region of interest pooling layer.
- Final fully connected layer and softmax layer are replaced by twin softmax layers and a bounding box regressor.

Improveed R-CNN: All 2000 candidate region proposals do not need to pass into CNN architecture; convolution operation is performed only once per image to generate a feature map. The entire network is combined and trained together hence decreasing the need for additional disk memory during training.

##### Faster R-CNN

Although Fast R-CNN achieved some improvements compared to R-CNN, it still suffers from a high computational cost that discourages its employment for real-time applications. The leading cause of the computational load finds its roots in the selective search algorithm. Ren et al. [144] designed Faster R-CNN attempts to overcome the mentioned issue building on two networks: Region Proposal Network (RPN) and Object Detection Network. The backbone of this architecture is a CNN which is common between object detection network and region proposal network. The authors of Faster R-CNN experimented with ZFNet and VGG-16 as the architecture’s backbone. The feature map produced by the backbone layer inputs the region proposal network to build anchors generated by sliding window convolution. The detection network of Faster R-CNN works similarly to Fast R-CNN. An ROI pooling layer is introduced to extract fixed-size region proposals. Twin softmax classifier and bounding box regressor are used for predicting and detecting objects of interest.

##### Region Based Neural Networks for Mammogram Analysis

Ribli [129] proposed a Faster R-CNN-based CAD system to detect and classify mammogram lesions. The method is compared to state-of-the-art techniques in the domain on the INBreast dataset. This model achieved second place in the DREAM Challenge for Digital Mammography, with an AUC value of 0.85. Authors also have made source code available to https://github.com/riblidezso/frcnn_cad (accessed on 1 June 2021). Xiao et al. [122] focused their efforts on making effective interlayer features and supporting improvements on detection capability. The authors used a novel attention module called CSABlock of the cascade R-CNN model. An adaptive pre-training strategy added to improve the detection capability further. A method for bilateral mass detection was performed by Zhang [123] using two different approaches such as supervised learning and Siamese-Faster-RCNN. To find regions of interest, authors have used morphological features and threshold segmentation. A network of supervised learning retrieves spatial transformation between the bilateral areas of the breast. Once these regions are registered, Faster RCNN performs mass detection. Preliminary results showed that bilateral analysis performs well for mass detection on single mammograms.

An automated method for mass detection in breast mammogram was developed by Agarwal et al. [124]. The technique relies on a Faster R-CNN model. Agarwal et al. first benchmarked their method on the OPTIMAM Mammography Image Database (OMID). When compared to the state-of-the-art techniques, their approach showed higher accuracy rates. Architectural distortion is one of the possible pre-malignant indications of breast cancer that can be missed out due to oversight errors. Ben-Ari et al. [125] developed a model to detect architectural distortion with an R-CNN based model. The method is tested on the DDSM mammogram dataset compared with another region-based network called faster RCNN and other previous work. A faster RCNN–based method to locate and classify breast lesions was presented by Zhang et al. [126], where mages are preprocessed to obtain better quality results. These images are then passed to five different feature extractors. These five models are used to see the difference in the Faster RCNN for the classification process. Finally, the regional suggestion network (RPN) is used to obtain detected regions. The results of simulations have proved that Inception ResNet V2 gives better performance as a feature extractor than the rest four models. Detecting multiple lesions contained in single mammograms is still an open issue. Most of the current literature consists of methods dealing with detections of single abnormalities in mammograms. Bhatti et al. [127] presented in dealt with multiple detection and segmentation of lesions for precise diagnosis. A ROI-based CNN called mask RCNN along with the feature pyramid network was used by the researchers. Bhatti et al. [127] extracted semantic features of an image on different resolution scales with the feature pyramid network (FPN). This model was trained and tested on two public datasets and achieved an overall accuracy rate of 91% when compared to other detection and segmentation architectures. Fan et al. [128] developed a computer-aided detection system for breast mass detection. It relies on Faster RCNN, and the experiments were conducted on a private mammogram dataset. The input images were taken using digital breast tomosynthesis (DBT). Authors have proposed CNN architecture with a region proposal network called RPN. This network generates region proposals in bounding boxes and a likelihood score of mass for each input slice. A free-response receiver operating characteristics (FROC) curve evaluates model performances.

#### 4.4.2. Unsupervised Deep Learning

Supervised Deep Learning approaches use previously labelled images to train models for classification and detection purposes. Among the many DL architectures, deep convolutional neural networks are heavily reliant on a high number of images to avoid the so-called overfitting during training steps [145]. The biomedical imaging domain lacks publicly available datasets containing manually labelled images. As described by Samala et al. [146], Data Augmentation and transfer learning techniques allow for overcoming the limitations mentioned above. The employment of pre-trained DL networks and their fine-tuning over a new application domain (mammogram images in our case) with the Transfer Learning paradigms are pretty standard for detecting suspicious regions in mammograms. Unsupervised deep learning approaches build on a training set of unlabelled images. The first objective is to learn new data representations, for example, by detecting a feature that makes it easier to extract some regions from images. The second is to learn a generative model as a probability distribution [145].

##### Autoencoders

Autoencoder is an unsupervised approach based on artificial neural networks. It learns how to compress and encode input data and, then, it rebuilds the data back from those encoded. Due to its design, it often reduces the data dimensionality while ignoring data noise. According to Baldi [15], autoencoders help to optimise lower-dimensional encoding by reducing the rebuilding error. Out of various forms of this approach, stacked autoencoders (SAEs) are used mainly for end-to-end learning to extract deep features from an image and segmentation. Stacked autoencoders can extract illustrative hidden patterns from the input image. This network addresses the problem of high variation in breast mass appearance or mass shape. (Stacked denoise autoencoder) SDAE-based models can reduce the inaccuracy of systems based on image processing being a noise-tolerant technique [147]. Kallenberg et al. [135] proposed a method to learn feature hierarchy from unlabelled data. This model is a convolutional sparse autoencoder (CSAE), a sparse autoencoder within the CNN structure. The main objective of this model is to segment breast density and obtain a risk score by acquiring features from unlabeled data. Afterwards, Kallengberg et al. trained a classifier to map those features to the labels of attention. The results showed that the density score has a robust connection with manual ones and that the method can be applied to any other segmentation task. For breast cancer detection, radiologists prefer to use both MLO and CC views to check the presence of lesions in mammograms. In 2016, Yang et al. [136] introduced a method based on sparse autoencoder to perform asymmetry analysis employing bilateral mammograms. The authors ran preprocessing steps on both the image sides and flipped the other side of the image in the same direction with the reference image. A shape context-based region matching method retrieved corresponding areas in images. Finally, the authors calculated similarities between the reconstructed vector and contralateral feature vector and got favourable results. This model produces a reconstruction vector by taking one side of bilateral regions as input. Finally, the similarity is calculated between the output regions of the model and matching input regions. A method to automatically generate feature sets from unlabelled and labelled data was presented by Petersen et al. [137]. It is an unsupervised approach called denoising autoencoder that accomplishes the contextual segmentation of an image. This method scores the density of breast of around 85 mammograms. It was shown that the proposed score correlates well with techniques of manual scoring, such as BI-RADS and Cumulus. An unsupervised deep learning-based technique was used by Selvathi and Poornila [138]. Stacked autoencoder and softmax classifier were combined together to form a deep network in this work. Four hidden layer autoencoders are used in the model. Mammograms are first preprocessed to remove artefacts and background noise to make the deep learning model more effective. After segmenting the region of interest, a deep network is used to detect cancer and calculate the density score. The model was tested on the MIAS dataset and achieved an accuracy rate of 98.5%. An integrated system with sparse autoencoder (SAE) and ML classifiers, such as decision tree, KNN, SVM and random forest, was developed by Selvathi and Poornila [139]. The SAE learns representations of features from images and classifiers cascaded with SAE and classification based on extracted features. The authors presented a comparative analysis of all these classifiers and showed that random forest gives the highest accuracy for the classification process. For mammography classification, a method for reducing and transforming features was presented by Taghanaki et al. [140]. Non-linear multi-objective autoencoders are used to change and reduce the total number of features to minimise feature reconstruction and classification errors. This method achieved a classification accuracy rate of 98.45%.

##### Generative Adversarial Networks (GAN)

**Figure 10:**
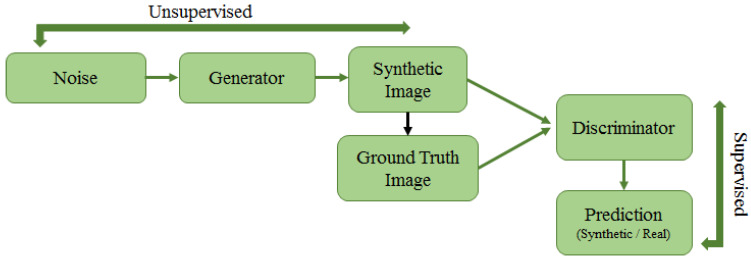
Architecture of GAN. The GAN comprises two networks: generator and discriminator. Generator synthetically generates images by adding noise, this is an unsupervised task. Discriminator tries to find whether images generated by the generator are real or fake as a supervised task. Both these networks compete against each other and gradually learn to produce better results.

Goodfellow et al. [148] proposed GANs (Generative Adversarial Networks) in 2014. The researchers widely adopt generative networks for data augmentation and sample generation [149]. Hussain et al. [150] examined GANs for data augmentation and generated mammogram datasets. The architecture of GAN consists of two networks: generator and discriminator. The generator helps to synthesize good quality images, and these images try to deceive the discriminator model trained on ground truth data. There are multiple variants of GAN. Figure 10 presents the basic architecture of GAN for generating and validating synthetic images. A GAN variant was proposed by Radford et al. [151]. The authors added batch normalization and discarded hidden layers that are fully connected. Singh et al. [152] proposed conditional generative adversarial network (cGAN) to segment mass from mammograms’ regions of interest. This model learns to represent masses for the creation of binary masks. The discriminative model discriminates masses from binary masks. The proposed model can also classify detected masses into four different categories: round, oval, lobular, and irregular. Class conditional GAN was proposed by Wu et al. [130] to achieve contextual in-filling to synthesize lesions onto the original input mammogram. The authors showed that GAN generates good quality synthetic images. Data scarcity and class asymmetrical distribution are two significant challenges in the biomedical imaging domain. Wu et al. [131] proposed a data augmentation method relying on a U-Net based model. They used semi-supervised learning and self-attention components to synthesize lesions onto healthy mammogram patches and to remove lesions from patches where they were present. Another similar method for the generation of different images of masses and using contextual infilling was presented by Shen et al. [132]. The method pairs feature descriptors from actual suspicious regions and the matching binary masks so the generator can extract matching shape, context details and boundaries. Experiments were conducted on DDSM and private datasets. The results show a 5.03% of improvement in detection rates when using the augmented dataset over original mass images. Korkinof et al. [134] proposed a method using progressively trained GAN to generate realistic and high-resolution synthetic mammograms. The model can generate high resolutions images, namely 1280 × 1024 pixels, which is the highest resolution achieved for medical image synthesis. Types of failures in the image generations are also reported in the paper. A system to insert and remove malignant features on mammograms was proposed by Becker et al. [133]. In their work, Becker et al. further determined whether human expert readers can easily understand whether the images were AI-generated. The system’s performance was evaluated and compared with three experts in the field to rate abnormality and the probability of an image being modified. However, the proposed solution represents a substantial trade-off between high-quality image generation and percentages of artefacts in themselves. The code and toy dataset has also been made available by the author at github.com/BreastGAN/experiment1 (accessed on 1 June 2021).

#### 4.4.3. Pros and Cons of Deep Learning Approaches

This subsection outlines some considerations about the pros and cons of supervised deep learning techniques such as FCNs and region-based neural networks and unsupervised deep learning methods, such as GANs and autoencoders. In Table 5, the methods mentioned above are listed according to techniques, tasks, datasets and performance metrics. AlGhamdi et al. [121] showed remarkably high accuracy, specificity and sensitivity rates, respectively, 91.47%, 92.01% and 91.22%, on DDSM in calcification detection with Dense-Unet FCN. Conversely, Unet in calcification detection achieved only 70.3% sensitivity on DDSM. Among FCN-based methods, the technique of Xu et al. [117] proved to have remarkably high inference knowledge capabilities scoring 0.91 dice similarity coefficient on two different datasets (DDSM, INBreast) in mass segmentation. Bhatti et al. [127] showed reasonably accurate performances of the Mask RCNN-FPN method on DDSM and INBreast in detection and segmentation of breast lesions (91% accuracy). Overall, both FCNs and RCNNs reach high accuracy rates in the detection and segmentation of masses. Being them supervised deep learning approaches, they go through a training step that plays a critical role in their performances. Concerning the unsupervised deep learning approaches, the performance of autoencoders and GANs is mainly benchmarked here against some particular tasks, such as mammogram image synthesis, mass detection, mass segmentation. Furthermore, some of them are paired with FCNs or classifiers to accomplish some specific tasks. The methods of We et al. [130,131] rely on GAN to perform data augmentation; then, the data are ingested by ResNet and Unet for classification. In both cases, the combination of the two techniques showed accurate performances on DDSM (0.896 in AUC) and OMID (0.846 in AUC). Korkinof et al. [134] opted for GAN to generate mammogram images, but they did not provide benchmarks for comparisons to other state-of-the-art techniques. The same consideration goes for CycleGAN, adopted by Becker et al. [133] for mammogram synthesis. Sparse autoencoders are used by Yang et al. [136] to analyse breast asymmetries (sensitivity 97%) on a local dataset. However, they did not account for specificity and false positives rates. On the other hand, sparse autoencoders are noticed to lack accuracy in breast density analysis. In greater detail, Kallenberg et al. [135] scored 0.59 on AUC; Petersen et al. used denoised sparse autoencoders, obtaining 0.62 AUC on a private set of data. The autoencoder-based methods by Selvathi and Poornila [138,139] and Taghanaki et al. [140] performed remarkably well in mammogram classification. For example, Selvathi and Poornila [138] achieved 98.50% accuracy on MIAS using stacked autoencoders. Furthermore, they improved by 0.39% when they combined sparse autoencoders and random forest [139] and ran it over MIAS. Taghanaki et al. [140] reached 98.45% accuracy on two different datasets, INBreast and IRMA, using a multi-objective optimisation of deep auto-encoder networks. The autoencoder optimises mean squared reconstruction error (MRE) and mean classification error (MCE). It appears to be a significant step forward in developing AI methods without the training step needed.

## 5. Discussion

This study surveys several scientific articles on suspicious regions detection in mammograms using a bottom-up approach, spanning low-level image feature-based techniques to deep learning techniques. One of the main points of this work is to analyse different approaches under three central perspectives: feature extraction, architectures used, and datasets employed to carry out experiments to detect and/or classify suspicious regions in mammograms.

### Final Points

- This paper surveys methods and techniques tackling the detection of suspicious regions in mammograms. The narrative of this work is bottom-up, spanning low-level image feature-based approaches to deep learning architectures. The paper provides summaries of different approaches in tables. In Table 2, Table 3, Table 4 and Table 5, a thorough description of features, performed tasks, datasets, performances is given for the aforementioned methods. Most approaches tackle mass detection and classification, while others address mammogram enhancement, microcalcification detection, and mammogram image generation with unsupervised deep learning architectures. Missing rates on datasets do not allow comparing some methods’ performances. Both MIAS and DDSM datasets stand out in the tables because their employment is far higher than others.
- Machine learning methods are reliable on most datasets. A method based on textural and shape features and K-means [45] achieves sensitivity rates higher than 94% on both datasets; a technique [44] relying on local contour features, 1D signature contour subsection and SVM shows an accuracy rate of 99.6% on a subset of DDSM. Elmoufidi et al. [50] obtained 96% of accuracy on MIAS using a swarm optimisation algorithm for heuristic parameter selection. The method in [40] adopts morphological features for mass detection in mammograms and achieves 92% of sensitivity, but no performance metrics are given about false positives. Geostatistical and concave geometry (alpha shapes) features [52] allow achieving high detection rates on MIAS (97.30%) and DDSM (91.63%). An LBP (local binary pattern) based method [58] turns out to be quite reliable for mass classification in MIAS (99.65% sensitivity and 99.24% specificity). A morphological top-hat transform method [61] is successful in mass and microcalcification detection on MIAS with around 99% specificity and sensitivity rates (Table 2). As highlighted in the pros and cons sections, when low-level image feature descriptors feed into deep neural networks, as in the method by Utomo et al. [70], they can achieve remarkably well (100% specificity and sensitivity rates) on MIAS. The same is true for methods relying on BoF (Bag of Features) and SVM, meaning they are discriminative features for mass classification in mammograms (DDSM). Accuracy rates are achieved by Deshmuk and Bhosle [75] on MIAS (92.3% accuracy) and DDSM (96.8% accuracy) by using an optimised SURF descriptor.
- As listed in Table 4, machine learning methods show some remarkable differences with methods in Table 2 and Table 3. Clustering-based methods by Kamil et al. [101] and Ketabi et al. [102] cannot achieve accuracy rates higher than 94% on MIAS and 90% on DDSM. Sharma et al. [113] achieved high performances in mass detection and classification on IRMA (specificity 99% and sensitivity 99%) and DDSM (specificity 96% and sensitivity 97%) using SVM. The ANN method proposed by Mahersia et al. [98] achieved an average mass recognition rates of 97.08% on MIAS.
- Deep learning methods (Table 5) raise the bar, exploiting their inference knowledge capabilities on more than a single dataset. The autoencoder-based method by Taghanaki et al. [140] performed mammography classification with 98.45% accuracy on INBreast and IRMA. The methods of Selvathi et al. [138,139] scored around 99% accuracy on MIAS by leveraging stacked autencoders, and sparse autoencoder plus random forest.
- Bruno et al. [29] highlighted how convolutional neural networks’ performance could be affected with noise and bias embedded with training dataset images. The availability of larger sized datasets might fully unleash the inference knowledge capabilities of deep learning architectures. Furthermore, it would enable a training-from-scratch process for neural networks. Further comparisons could be then carried out with pre-existing DL models that are fine-tuned over a limited sized mammogram dataset using transfer learning. It is necessary to highlight that most deep learning methods in the biomedical imaging field currently adopt the above-mentioned pipeline laying on data augmentation plus transfer learning, due to the lack of publicly available and manually annotated datasets.
- The good performances in mammogram synthesis obtained by Becker et al. [133] and Wu et al. [130,131] open new perspectives to the generation of larger mammogram datasets.

## 6. Conclusions

Image processing and artificial intelligence have progressed and expanded significantly in the medical field, especially diagnostic imaging. These advancements have greatly influenced computer-aided diagnosis (CAD) systems to detect and/or classify suspicious regions from mammograms. This study wants to represent a comprehensive insight into various approaches based on low-level image features, machine learning, and deep learning by comparing them on publicly available datasets. The performance of these approaches guides researchers in this domain to select an appropriate method for their applications. Computational models based on these approaches generally represent the core of CAD (computer-aided diagnosis) systems, suggesting regions of interests and leaving last words to medical doctors and practitioners. In this section, concise replies to the questions raised at the beginning of the paper are provided and described as follows:

(1) Shape-based, texture-based and local keypoint descriptors are the most common techniques used to extract low-level image features from mammograms;
(2) Machine learning approaches such as SVM, ANN, and various clustering techniques are also quite successful over various medical imaging tasks, especially to detect/classify abnormality from mammograms;
(3) Both supervised and unsupervised DL approaches have proven to be best for various mammogram analysis tasks;
(4) As listed in Table 1, researchers in the community of biomedical imaging ran experiments on different publicly available and commonly cited datasets such as SureMaPP, DDSM, INBreast, BCDR, IRMA, BancoWeb LAPIMO etc. Each dataset features images with several properties, due to different acquiring device properties.

Much work has already been done for computer-aided breast cancer detection, out of which few studies are already implemented and transformed into commercial products. Due to the lack of big sized publicly available datasets with manual annotations, the current deep learning architectures cannot fully unleash their inference knowledge capabilities for other tasks, such as object detection, classification and segmentation. Unsupervised learning techniques, such as GANs and autoencoders, appear to be promising solutions to fill the dimensionality gap between biomedical imaging and other common computer vision topics.

## Data Availability

Not applicable.

## Abbreviations

The following abbreviations are used in this manuscript:

CAD	Computer-Aided Diagnosis
BI-RADS	Breast Imaging Reporting and Database System
AI	Artificial Intelligence
ML	Machine Learning
DL	Deep Learning
SVM	Support Vector Machine
ANN	Artificial Neural Network
CNN	Convolutional Neural Network
SIFT	Scale Invariant Feature Transform
SURF	Speed Up Robust Feature
FCN	Fully Convolutional Network
RCNNN	Region-Based Convolutional Neural Network
GAN	Generative Adversarial Network
MLO	Mediolateral Oblique
CC	Craniocaudal
ROI	Region of Interest
kNN	k-Nearest Neighbour
MC	Microcalcification
MCL	Multiple Concentric Layers
MRE	Mean Squared Reconstruction Error
MSE	Mean Squared Error
GLCM	Gray-Level Co-occurrence Matrix
GLRLM	Gray-Level Run-Length Matrix
LBP	Local Binary Patterns
LQP	Local Quinary Patterns
CLAHE	Contrast Limited Adaptive Histogram Equalization
BRIEF	Binary Robust Independent Elementary Features
SOM	Self Organising Maps
GA	Genetic Algorithms
PFCM	Possibilistic Fuzzy C-Means
MIAS	Mammographic Image Analysis Society
DDSM	Digital Database of Screening Mammography
CBIS-DDSM	Curated Breast Imaging Subset-DDSM
BCDR	Breast Cancer Digital Repository
